# A Phase I Dose Escalation Study of the Triple Angiokinase Inhibitor Nintedanib Combined with Low-Dose Cytarabine in Elderly Patients with Acute Myeloid Leukemia

**DOI:** 10.1371/journal.pone.0164499

**Published:** 2016-10-07

**Authors:** Christoph Schliemann, Joachim Gerss, Stefanie Wiebe, Jan-Henrik Mikesch, Nicola Knoblauch, Tim Sauer, Linus Angenendt, Tobias Kewitz, Marc Urban, Trude Butterfass-Bahloul, Sabine Edemir, Kerstin Vehring, Carsten Müller-Tidow, Wolfgang E. Berdel, Utz Krug

**Affiliations:** 1 Department of Medicine A, University Hospital Muenster, Muenster, Germany; 2 Institute of Biostatistics and Clinical Research, University Hospital Muenster, Muenster, Germany; 3 Centre for Clinical Trials, University Hospital Muenster, Muenster, Germany; University of Maryland Baltimore County, UNITED STATES

## Abstract

Nintedanib (BIBF 1120), a potent multikinase inhibitor of VEGFR-1/-2/-3, FGFR-1/-2/-3 and PDGFR-α/-β, exerts growth inhibitory and pro-apoptotic effects in myeloid leukemic cells, especially when used in combination with cytarabine. This phase I study evaluated nintedanib in combination with low-dose cytarabine (LDAC) in elderly patients with untreated or relapsed/refractory acute myeloid leukemia (AML) ineligible for intensive chemotherapy in a 3+3 design. Nintedanib (dose levels 100, 150, and 200 mg orally twice daily) and LDAC (20 mg subcutaneous injection twice daily for 10 days) were administered in 28-day cycles. Dose-limiting toxicity (DLT) was defined as non-hematological severe adverse reaction CTC grade ≥ 4 with possible or definite relationship to nintedanib. Between April 2012 and October 2013, 13 patients (median age 73 [range: 62–86] years) were enrolled. One patient did not receive study medication and was replaced. Nine (69%) patients had relapsed or refractory disease and 6 (46%) patients had unfavorable cytogenetics. The most frequently reported treatment-related adverse events (AE) were gastrointestinal events. Twelve SAEs irrespective of relatedness were reported. Two SUSARs were observed, one fatal hypercalcemia and one fatal gastrointestinal infection. Two patients (17%) with relapsed AML achieved a complete remission (one CR, one CRi) and bone marrow blast reductions without fulfilling PR criteria were observed in 3 patients (25%). One-year overall survival was 33%. Nintedanib combined with LDAC shows an adequate safety profile and survival data are promising in a difficult-to-treat patient population. Continuation of this trial with a phase II recommended dose of 2 x 200 mg nintedanib in a randomized, placebo-controlled phase II study is planned. The trial is registered to EudraCT as 2011-001086-41.

***Trial Registration*:** ClinicalTrials.gov NCT01488344

## Introduction

Acute myeloid leukemia (AML) is a clonal malignant disorder that results from genetic and epigenetic changes in pluripotent stem or more differentiated progenitor cells [1]. It is predominantly a disease of the elderly patient, with a median age at diagnosis of 70 years [2]. The clinical course of AML is heterogeneous and response to therapy and outcome are influenced by several patient- and disease-related features, including age, general performance status, comorbidities and cytogenetic aberrations [3, 4]. Older patients more frequently present with a combination of unfavorable risk factors, including adverse cytogenetics and a higher frequency of secondary AML arising from antecedent hematological disorders, rendering their disease particularly difficult to treat. Of patients over 60 years of age only 25% survive two or more years, even after intensive induction chemotherapy [5]. Consequently, there is an unmet need for novel treatment strategies that provide efficacy and favorable tolerability for older patients with AML who are unable to tolerate intensive chemotherapy regimens.

Nintedanib (BIBF 1120) is a potent, oral, small-molecule tyrosine kinase inhibitor of VEGFR-1/-2/-3, FGFR-1/-2/-3 and PDGFR-α/-β signaling [6], and has been recently approved for second line treatment of non-small cell lung cancer (NSCLC) of adenocarcinoma histology in combination with docetaxel. All three signaling pathways have been shown to play critical roles in the regulation of angiogenesis, not only in solid tumors but also in hematological malignancies such as AML [7]. Secreted by leukemic blasts, VEGF, bFGF and PDGF act in a paracrine fashion on the bone marrow vasculature and stroma, which in turn promote leukemia cell survival and growth [8, 9]. Interestingly, these ligand-receptor pairs are also involved in autocrine signaling loops, in which leukemia-derived growth factors directly support the growth of VEGFR- [10–12], FGFR- [13–15], and PDGFR- [16] expressing leukemic cells. In preclinical experiments, BIBF 1120 displayed potent growth inhibitory and proapoptotic effects in various AML cell lines in the nanomolar range [17].

While the antibody-mediated pharmacological inhibition of single angiogenic mediators in AML has been largely disappointing so far (e.g., bevacizumab) [18, 19], nintedanib offers the theoretical advantage of simultaneously targeting multiple signaling pathways of pathophysiological relevance, including both paracrine crosstalks between AML cells and vascular cells and autocrine signaling loops in leukemic cells themselves. In addition, the compound exhibits activity against the Src family members Src, Lyn and Lck as well as against Flt-3 [6], and thus could represent a particularly attractive drug for AML therapy.

Here, we report the results of a phase I dose escalation trial of nintedanib combined with low-dose cytarabine (LDAC) in elderly patients with untreated (de novo and secondary), relapsed or refractory AML not considered eligible for intensive chemotherapy.

## Methods

### Patients and eligibility

Elderly patients (≥ 60 years) with de novo, secondary, or previously treated AML (except acute promyelocytic leukemia) according to the French-American-British (FAB) or World Health Organization (WHO) classification not considered fit to receive intensive induction or salvage chemotherapy were eligible for the study. Cytogenetics and *NPM1*, *FLT3* and *CEBPA* mutational status and assignment to risk groups according to the European LeukemiaNet (ELN) classification were done according to standard procedures. In patients with 20–30% bone marrow blasts, the indication for 5-azacitidine had to be considered prior to inclusion into the trial. By amendment of March 2013, patients at risk for hollow organ perforation (i.e. patients with ulcerative colitis, Crohn`s disease, or diverticulitis) could only be enrolled if the potential benefit of the study participation outweighed the risk for perforation in the opinion of the investigator. Exclusion criteria included: known central nervous system manifestation of AML; inadequate liver function (ALT and AST ≥ 2.5 x ULN) unless caused by leukemic infiltration; known chronically active hepatitis C infection or acute hepatitis; chronically impaired renal function (creatinine clearance < 30 ml/min); uncontrolled hypertension with a resting systolic blood pressure > 160 mmHg or diastolic blood pressure > 95 mmHg despite adequate treatment; severe trauma or surgery within 4 weeks of study entry; severe, non-healing wounds, ulcers or fractures; uncontrolled active infection; concurrent malignancies other than AML or other severe diseases which in the opinion of the investigator were likely to influence the endpoint assessment; hypersensitivity to cytarabine (not including drug fever or exanthema); parallel participation in another clinical trial for the same indication; any severe concomitant condition, which made it undesirable for the patient to participate in the study or which could jeopardize compliance with the protocol. Written informed consent was obtained from all patients before study enrolment.

### Study design and treatment

The trial protocol, the CONSORT diagram and the TREND checklist are available as supporting information (see S1 and S2 Protocols, S1 Fig and S1 Checklist). This was a prospective, single-center, dose escalation phase I study to assess the safety and tolerability of nintedanib combined with LDAC in elderly patients with de novo or relapsed or refractory AML unfit for intensive chemotherapy. The study was approved by the Ethics Committee of the Physicians Chamber of Westfalia-Lippe and the University of Muenster, Germany, and conducted in accordance with the Declaration of Helsinki and Good Clinical Practice. The trial was registered to www.clinicaltrials.gov (identifier NCT01488344) and to EudraCT as 2011-001086-41.

**Fig 1:**
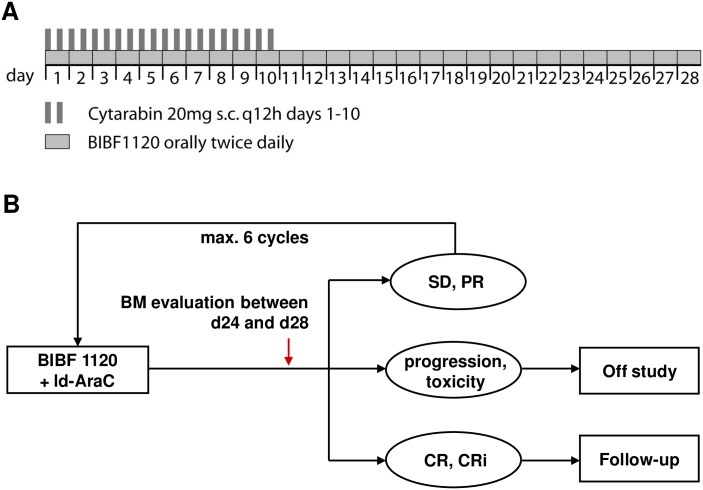
Study overview. (A) LDAC was administered at 20 mg twice daily subcutaneously on days 1 to 10 of a 28-day cycle. Nintedanib was taken orally twice daily at three dose levels (100 mg, 150 mg, and 200 mg twice daily). Dose escalation was performed in a classical 3+3 design. (B) Patients received up to 6 cycles of combination therapy until disease progression or achievement of a CR.

Dose escalation was performed in a classical 3+3 design with 3 predefined dose levels of nintedanib (100 mg twice daily for 28 days of a 28-day cycle in dose level (DL) 1, 150 mg in DL2, and 200 mg in DL3) and patients were entered in cohorts of 3–6. Since a maximum tolerable dose (MTD) for nintedanib in combination with various chemotherapeutic agents had already been evaluated in clinical trials in solid tumors, an accelerated dose escalation to the recommended dose of nintedanib in combination with chemotherapeutic agents (2 x 200 mg) was chosen for the combination with LDAC. LDAC was administered from days 1–10 at 20 mg twice daily by subcutaneous injection (Fig 1A). Toxicity was assessed by Common Terminology Criteria for Adverse Events (CTCAE) version 4.0 criteria. Patients evaluable for safety analysis had to have received at least one dose of nintedanib and LDAC. The determination of the MTD was based on the occurrence of dose limiting toxicities (DLTs) in the first treatment cycle. In the absence of DLTs in the first cycle, dose escalation of nintedanib proceeded to the next DL. In case of one DLT, up to 3 further patients were recruited into the same DL. The highest DL with ≤ 1 DLT out of 6 patients was defined as the maximum tolerable dose (MTD) and set as the phase II recommended dose (P2RD). In case a DL was considered for phase II with less than 6 patients evaluated (i.e. no DLT in 3 patients), further patients were recruited to reach a total of 6 evaluable patients. A DLT was defined as every severe adverse reaction CTC grade IV with possible or definite relationship to nintedanib. Designated exceptions for DLT determination included therapy-related cytopenias as signs of an intended anti-leukemic activity and cytopenia-associated complications (neutropenic fever, neutropenic infections, and thrombocytopenic bleedings). However, these complications could constitute a DLT if occurring in an unexpected high frequency, as judged by the DMC. Furthermore, complications such as neutropenic infections, thrombocytopenic bleedings, deterioration of the general condition, laboratory abnormalities, death, tumor lysis syndrome, or organ failure, were also excluded from DLT determination, whenever these were clearly attributable to AML progression in the judgment of the investigator. Adverse events (AE) of special interest (AESI) were elevation of AST and/or ALT > 3x upper level of normal (ULN) combined with an elevation of bilirubin > 2x ULN, and any hollow organ perforation occurring after the first intake of study medication.

A bone marrow evaluation was done at the end of each cycle between days 24 and 28. Cycles were scheduled every 4 weeks until progressive disease (PD), intolerance/toxicity, discontinuation upon patient or investigator request, or achievement of a complete remission with or without peripheral blood count recovery (CR or CRi) (Fig 1B). Patients with a partial remission (PR) or stable disease (SD) continued treatment for up to 6 therapy cycles.

### Endpoints

The primary objective was to evaluate the safety and tolerability of nintedanib combined with LDAC to establish a P2RD. Secondary objectives were treatment response and overall survival (OS).

### Biomarker evaluation

Serum levels of VEGF and soluble VEGF receptor 2 (sVEGFR-2) were measured before treatment initiation and after each therapy cycle by enzyme-linked immunosorbent assays according to the manufacturer`s instructions (R&D Systems, Minneapolis, USA).

### Statistical analysis

Baseline disease characteristics and demographic data of all patients were summarized descriptively, using medians, means and ranges. All patients who received at least one dose of nintedanib and LDAC were included into the analysis of safety, tolerability and efficacy. OS was measured from the first day of treatment until death of any cause and was estimated using the Kaplan-Meier method. Correlations between serum angiogenic mediators were assessed using the Pearson correlation coefficient. The Wilcoxon matched-pair signed rank test was used to compare levels of angiogenic factors in individual patients at diagnosis and following study treatment. Two-sided *P* values lower than 0.05 were considered to indicate significant differences. All analyses were performed using the SPSS Statistics software package, version 22.0 (IBM Corp., Armonk, NY, USA).

## Results

### Patients

Between April 2012 and October 2013, 13 patients with a median (range) age of 73 (62–86) years were enrolled (DL1 (n = 3), DL2 (n = 4), DL3 (n = 6)). One patient in DL2 did not receive study medication due to rapid AML progression and deterioration of general condition and was replaced. Table 1 summarizes the clinicopathological characteristics of all patients. The majority of patients (n = 9, 69%) had relapsed (n = 5) or refractory (n = 4) AML and had received one or more prior therapies (median (range) 2 (0–3)), including intensive chemotherapy regimens (n = 9), allogeneic hematopoietic stem cell transplantation (n = 1), 5-azacitidine (n = 1), and experimental compounds (n = 3). Four patients (31%) were enrolled with newly diagnosed AML (two with de novo and two with secondary AML). Median ECOG performance status (range) was 1 (1–3). According to ELN criteria, six patients (46%) had adverse risk, five (38%) had intermediate risk, and one patient (8%) had favorable cytogenetic / molecular risk (unknown in one patient). An internal tandem duplication in the fms-like tyrosine kinase 3 gene (FLT3-ITD) was found in one patient.

**Table 1:** Patient demographics and baseline disease characteristics.

	n = 13
**Median (range) age, years**	73 (62–86)
**Male/female, n**	7/6
**ECOG performance status, n**	
**0**	0
**1**	9
**2**	2
**3**	2
**Disease status, n**	
**untreated**	4
**de novo**	2
**secondary**	2
**relapsed**	5
**refractory**	4
**FAB classification, n**	
**M0/M1/M2**	6
**M4/M5**	5
**M6**	1
**unclassified**	1
**ELN risk, n**	
**favorable**	1
**intermediate**	5
**adverse**	6
**missing**	1
**Median (range) WBC count, x 10^9^/L**	2.71 (0.90–77.40)
**Median (range) peripheral blasts, %**	9 (0–87)
**Median (range) of bone marrow infiltration, %**	60 (20–80)
**Median (range) previous lines of therapy, n**	2 (0–3)

FAB: French-American-British classification for AML; ELN: European Leukemia Net; WBC: White blood cells

### Safety and tolerability

Four patients (33%) received one course of treatment, five (42%) received two, two (17%) received three, and one (8%) received six cycles. In total, five tablets of nintedanib were not taken in three patients, corresponding to a compliance rate of 99.6%. No DLTs were reported during dose escalation to DL3. Thus, three additional patients were enrolled at DL3 and 200 mg nintedanib twice daily was designated as the P2RD in combination with LDAC since no DLTs occurred in a total of six patients treated at DL3.

Overall, the safety profile of nintedanib was similar to that reported in previous studies [20]. The most common AEs of any grade associated with nintedanib treatment as determined by the investigators were gastrointestinal AEs, including diarrhea (n = 9), nausea (n = 7) and emesis (n = 6) (Table 2). Nintedanib-related grade ≥ 3 AEs were reported in four patients. Irrespective of relatedness, 17 grade ≥ 3 AEs were reported in 9 patients. The most common of which were febrile neutropenia and gastrointestinal disorders, which each occurred in two patients. A complete list of all AEs can be found in S1 and S2 Tables.

**Table 2:** Frequency of patients with nintedanib-related adverse events across all dose levels.

MedDRA preferred term (v15.1)	All grades^a^, n	Grade ≥ 3, n
Diarrhoea	9	1
Nausea	7	0
Vomiting	6	0
Constipation	3	0
Blood bilirubin increased	2	0
Dry mouth	2	0
Hypotonia	2	0
Pyrexia	2	0
Gastrointestinal infection	1	1
Hypercalcaemia	1	1
Hyperuricaemia	1	1
Abdominal distension	1	0
Abdominal pain	1	0
Abdominal pain upper	1	0
Asthenia	1	0
Alkaline phosphatase increased	1	0
Decreased appetite	1	0
Dysgeusia	1	0
Eructation	1	0
Headache	1	0

^a^Note: Data represent the highest CTCAE grade reported.

A total of 12 SAEs (coded with 17 MedDRA codes) were reported in nine patients, one at DL1, four at DL2, and seven at DL3. All were single events except neutropenic fever, which was reported 4 times in three patients and pyrexia, which occurred in two patients (Table 3). SAEs had to be reported until 28 days after last protocol treatment. Death due to acute myeloid leukemia progression was reported as SAE in one patient. One additional patient died from disease progression before start of therapy but after signing informed consent. No AESIs were observed in this study. Two suspected unexpected severe adverse reactions (SUSARs) were reported in two patients at DL2 and DL3, respectively. One patient developed a fatal gastrointestinal infection, and no fatality has been previously reported for gastrointestinal infection caused by nintedanib. The other patient experienced hypercalcemia due to unknown reasons in a phase of AML progression and died shortly after. Serum alkaline phosphatase and parathyreoid hormone were within the normal range in this patient, but further evaluation of hypercalcemia was not possible since the patient requested transferral to a hospice. Hypercalcemia had not been associated with nintedanib so far.

**Table 3:** SAEs, SARs, SUSARs and AESIs reported in the study (presented by relatedness to nintedanib, MedDRA codes for overall 12 cases, MedDRA v15.1).

MedDRA preferred term	n	Max. CTCAE grade	Dose level
**SAE**	**11**		
Febrile neutropenia	4	3	DL2, DL3
Pyrexia	2	2	DL1, DL3
Thrombocytopenia	1	4	DL2
Atrial flutter	1	3	DL3
Renal failure acute	1	3	DL3
Pneumonia	1	2	DL3
Acute myeloid leukemia^a^	1	5	DL3
**SAR**	**4**		
Diarrhoea	1	3	DL3
Vomiting	1	2	DL3
Nausea	1	2	DL3
Abdominal pain upper	1	2	DL3
**SUSAR**	**2**		
Hypercalcaemia	1	5	DL2
Gastrointestinal infection	1	5	DL3
**AESI**	**0**		
-	-	-	-

^a^This patient experienced lethal “Acute myeloid leukemia progression”, coded with MedDRA preferred term “Acute myeloid leukemia”

### Treatment outcome

Two out of 12 treated patients (17%) had an objective response to nintedanib combined with LDAC. A CR lasting one month was reported in one patient after three courses at DL1, with an additional patient achieving a CRi of four months after the first cycle at DL2. Both patients had relapsed disease after intensive chemotherapy when enrolled in the study. SD was reported in five patients, with three of them showing significant reduction of bone marrow blasts, and four patients experienced PD. The patient with fatal GI tract infection died before response evaluation (indeterminate cause). All patients responding with a CR/CRi or a blast reduction were FLT3-ITD negative.

**Fig 2:**
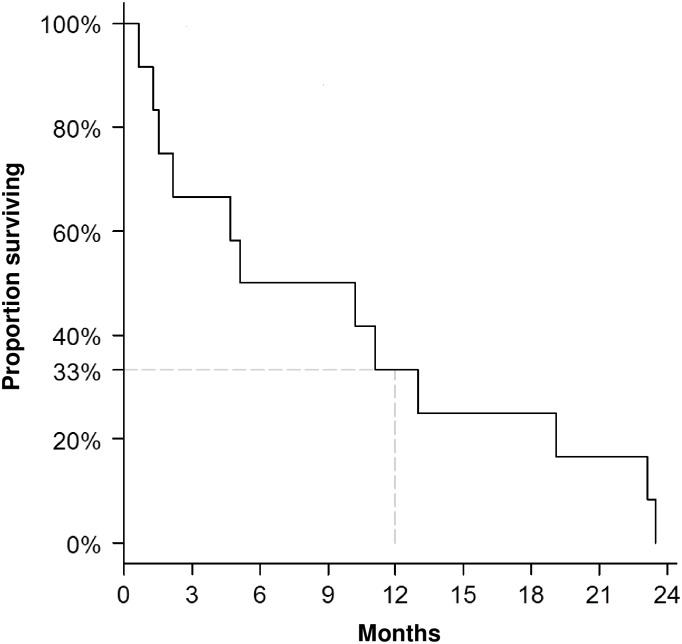
Kaplan-Meier curve of overall survival. Median OS of treated patients was 234 days, and one-year OS (95% CI) was 33.3% (10.3–58.8%).

All patients had died at the time of OS evaluation. Median OS of the treated study cohort was 234 days and 1-year survival was 33.3% (95% confidence interval (CI), 10.3–58.8%) (Fig 2). Interestingly, one patient who had persisting leukemia after two courses of nintedanib and LDAC at DL3 survived almost two years after start of therapy with best supportive care but without further specific anti-leukemic treatment.

### Serum levels of angiogenic mediators during treatment with nintedanib and LDAC

**Fig 3:**
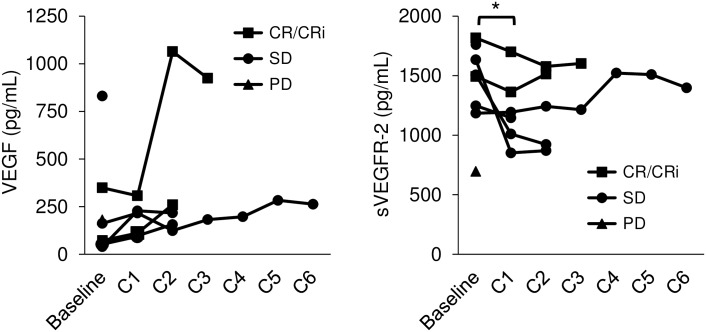
Changes in VEGF and sVEGFR-2 serum concentrations during therapy. While pre- and post-therapeutic levels of VEGF did not significantly differ (median 67.5 vs. 163.5 pg/mL, *P* = 0.173, Wilcoxon test), sVEFGR-2 serum concentrations significantly decreased after one cycle of study therapy (1500 vs. 1169.5 pg/mL, * *P* = 0.046).

Mean serum levels (range) of VEGF and sVEGFR-2 obtained from 8 patients at baseline were 117 pg/mL (40–831 pg/mL) and 1500 pg/mL (696–1817 pg/mL), respectively. VEGF levels positively correlated with sVEGFR-2 levels (r = 0.419, *P* = 0.042). In patients in whom post-therapeutic blood samples were available, sVEGFR-2 concentrations significantly decreased after the first cycle of therapy as compared to baseline (median 1500 vs. 1169.5 pg/mL, *P* = 0.046, Wilcoxon test), while VEGF did not significantly differ (67.5 vs. 163.5 pg/mL, *P* = 0.173) (Fig 3). However, there was no apparent relation between therapy-induced changes in VEGF or sVEGFR-2 levels and response to therapy.

## Discussion

This single-arm dose escalation trial was the first to investigate the safety and efficacy of the triple angiokinase inhibitor nintedanib added to standard LDAC therapy (400 mg over ten days) in elderly patients with newly diagnosed, relapsed or refractory AML. Using a classical 3+3 design no DLTs were reported and no safety concerns emerged from combination treatment. The dose level of 200 mg taken twice daily was established as the recommended phase II dose. In fact, 200 mg twice daily is the dose at which nintedanib has recently been approved for the treatment of advanced NSCLC in combination with docetaxel [20].

Overall, the toxicity profile of nintedanib observed in this study is consistent with previous large phase III studies of nintedanib as single-agent [21] or in combination with chemotherapy [20]. The only unexpected adverse reaction was a fatal hypercalcemia, besides a fatal outcome of an otherwise expected gastrointestinal infection. Since this hypercalcemia occurred in a patient with progressive disease, an association with disease progression within bone marrow is possible. As expected, gastrointestinal toxicities were the main nintedanib-related AEs reported. In fact, the orthogonal spectrum of side effects, with myelosuppression being the most relevant toxicity for LDAC [22] and with gastrointestinal adverse effects for nintedanib, should facilitate combinability between these two agents.

Elderly patients with newly diagnosed AML can expect a 13–19% chance of remission from treatment with single-agent LDAC [22–24], while there are no data available on the efficacy of LDAC monotherapy beyond first-line. In the study reported here, a CR rate of 17% has been observed in a cohort in which two-thirds of patients had relapsed or refractory AML and in which half of patients had unfavorable cytogenetics, thus representing a particularly difficult-to-treat elderly population. Interestingly, these two CRs were achieved in patients who were enrolled in the study with relapsed AML after intensive cytarabine-based induction chemotherapy. Furthermore, the 1-year OS rate of 33% observed in our cohort with the combination of LDAC and nintedanib is a promising preliminary finding, although caution has to be taken given the small cohort and the wide confidence interval. Given that the majority of patients entered the study in a relapsed or refractory situation, this would compare favorably with 1-year OS rates for single-agent LDAC in chemotherapy naïve patients of 22–25% [22–24], even though the most recent United Kingdom Medical Research Council (MRC) non-intensive AML studies in treatment-naïve patients indicate similar 1-year OS rates of approximately 30% with LDAC [25]. The reasons for the encouraging OS in the absence of a higher CR rate and a prolonged remission duration in our study are not obvious. Similar findings, however, have been made with demethylating agents, which are able to provide a survival advantage in elderly patients with AML not responding with a CR [26–28]. Interestingly, one patient treated with LDAC and nintedanib at the highest dose level survived almost two years after treatment initiation with persisting leukemia and best supportive care, but without further specific anti-leukemic therapy.

Bone marrow and circulating levels of VEGF and its receptors have been consistently shown to be increased in AML patients as compared to healthy individuals [10, 29, 30], indicating that the VEGF/VEGFR axis plays an important role in the pathophysiology of the disease [11, 12]. In the present study, treatment with nintedanib and LDAC was associated with a decrease of sVEGFR-2 serum concentrations, whereas blood levels of VEGF did not change significantly. Although the mechanisms responsible for the decrease of circulating VEGF receptors upon small molecule inhibition are not fully understood, our results are entirely in line with previous observations made with nintedanib in solid tumors [31] and with other kinase inhibitors targeting VEGFR-2 such as cediranib [32] or AG-01736 [33] in AML. Alternatively, the observed decrease in sVEGFR-2 levels could also be attributed to the acute reduction of the leukemic burden by LDAC. Thus, it remains to be seen whether sVEGFR-2 might serve as a valuable pharmacodynamic indicator of nintedanib drug exposure when used in combination with chemotherapy in AML.

In conclusion, adding nintedanib to LDAC at a RP2D of 200 mg twice daily was found to be safe in elderly patients with AML. Based on the encouraging survival data obtained in phase I, a randomized, placebo-controlled, multi-center phase II study evaluating the efficacy of the combination of nintedanib and LDAC is in preparation.

## Supporting Information

S1 Checklist TREND checklist. (PDF)

S1 Fig CONSORT diagram. (TIFF)

S1 Protocol Study protocol version 1.0. (PDF)

S2 Protocol Study protocol version 2.0 (amended protocol). (PDF)

S1 Table Complete list of adverse events which occurred in patients treated with nintedanib. (PDF)

S2 Table Frequency of nintedanib-treated patients with adverse events. (PDF)
