# Supplementary figures and images for: A Phase I Dose Escalation Study of the Triple Angiokinase Inhibitor Nintedanib Combined with Low-Dose Cytarabine in Elderly Patients with Acute Myeloid Leukemia
Source: PLoS One. 2016 Oct 7;11(10):e0164499. doi: 10.1371/journal.pone.0164499 (PMC5055288; doi:10.1371/journal.pone.0164499)

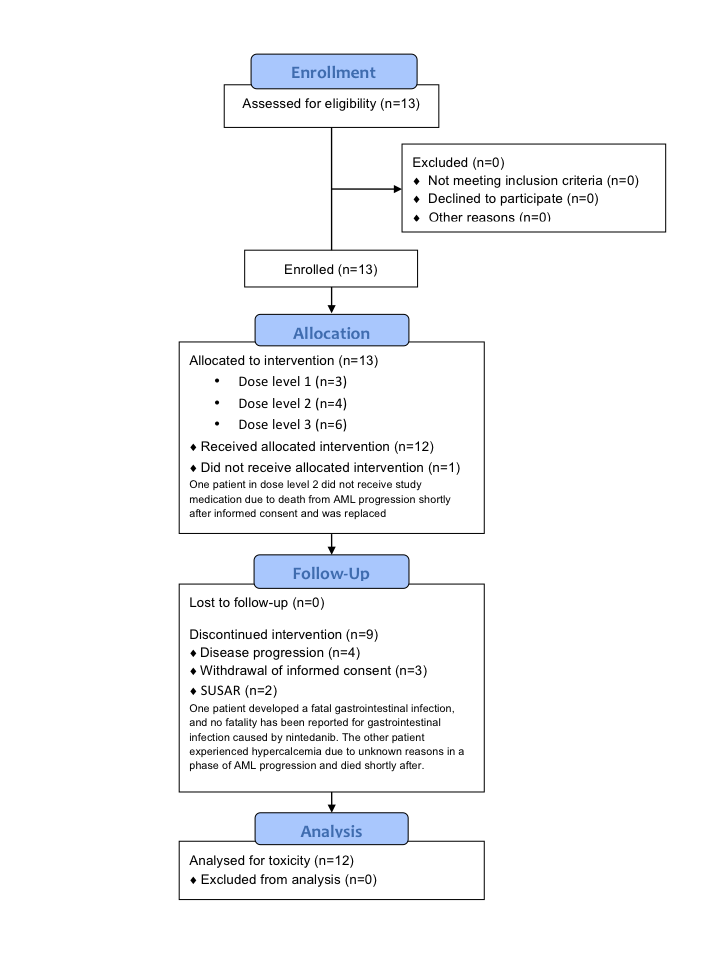

Supplement: S1 Fig — (TIFF) [file pone.0164499.s002.tiff]
