# Supplementary material for: A Phase I Dose Escalation Study of the Triple Angiokinase Inhibitor Nintedanib Combined with Low-Dose Cytarabine in Elderly Patients with Acute Myeloid Leukemia
Source: PLoS One. 2016 Oct 7;11(10):e0164499. doi: 10.1371/journal.pone.0164499 (PMC5055288; doi:10.1371/journal.pone.0164499)
Supplement: S1 Protocol — (PDF) [file pone.0164499.s003.pdf]

A single-arm, open label, multi-center phase I/II trial to assess the safety and efficacy of BIBF 1120 added to low-dose cytarabine in elderly patients with AML unfit for an intensive induction therapy

Study protocol

July 2011

Version 1.0.7

CONFIDENTIAL

**Sponsor:**	Universitätsklinikum Münster (UKM)
**Co-ordinating investigator:**	PD Dr. med. Utz Krug
**EudraCT Number:**	2011-001086-41
**Clinicaltrials.gov identifier:**	ausstehend
**WHO Universal Trial Number (UTN):**	U1111-1122-6147
**Protocol Code of the Sponsor:**	UKM10_0014
**Running Title:**	BIBF 1120 in AML

Sponsor: **Universitätsklinikum Münster**
Dr. rer. pol. Christoph Hoppenheit
Albert-Schweitzer-Campus 1, building D5
48149 Münster

____
(Date and Signature)

Co-ordinating investigator: **PD Dr. med. Utz Krug**
Universitätsklinikum Münster
Medizinische Klinik und Poliklinik A
Albert-Schweitzer-Campus 1, building A1
48149 Münster

____
(Date and Signature)

Statistician: **Dr. rer. nat. Joachim Gerß**
Universitätsklinikum Münster
Institut für Biometrie und klinische Forschung
Albert-Schweitzer-Campus 1, building A11
48149 Münster

____
(Date and Signature)

**Study physician**:	Dr. med. Nicole Knoblauch<br>Universitätsklinikum Münster<br>Medizinische Klinik und Poliklinik A<br>Albert-Schweitzer-Campus 1, building A1<br>phone: +49-251-83-45362<br>fax: +49-251-83-47599<br>email: nicole.knoblauch@ukmuenster.de
**Study Center**:	Universitätsklinikum Münster<br>Medizinische Klinik und Poliklinik A<br>Studiensekretariat: Claudia Tuschen<br>Albert-Schweitzer-Campus 1, building D3<br>phone: +49-251-83-52995<br>fax: +49-251-83-52673<br>email: claudia.tuschen@ukmuenster.de
**Study Drug Distributor:**	Boehringer Ingelheim GmbH und Co. KG<br>Binger Strasse 173<br>55216 Ingelheim am Rhein
**Monitoring:**	Zentrum für klinische Studien<br>Universitätsklinikum Münster<br>Von-Esmarch-Strasse 62<br>48149 Münster
**Data Monitoring Committee:**	Prof. Dr. med. Thomas Büchner, Münster<br>Prof. Dr. med. Claudia Rössig, Klinik für pädiatrische Hämatologie, Universitätsklinikum Münster<br>Dr. rer. nat. Achim Heinecke, Münster

TABLE OF CONTENTS

1 ABBREVIATIONS 8

2 BACKGROUND INFORMATION / INTRODUCTION 10

2.1 Condition background and current treatment 10

2.2 Investigational product background 11

2.2.1 Preclinical information 12

2.2.2 Clinical information 14

3 STUDY OBJECTIVES 18

3.1 Rationale for this study 18

3.1.1 Benefit / risk ratio 18

3.2 Study objectives 19

3.2.1 Phase I 19

3.2.2 Phase II 19

3.3 Study design 20

4 SELECTION, REGISTRATION AND WITHDRAWAL OF PATIENTS 20

4.1 Number of patients 20

4.2 Admission criteria 20

4.2.1 Inclusion criteria 20

4.2.2 Exclusion criteria 20

4.3 Patient registration 21

4.3.1 Registration 21

4.4 Withdrawal of patients 22

5 STUDY, STANDARD AND CONCOMITANT TREATMENTS 23

5.1 Investigational product 23

5.1.1 Relevant physical, chemical and pharmaceutical properties 23

5.1.2 Instructions for storage and handling 23

5.1.3 Interactions with concomitant medications 23

5.1.4 Drug accountability 23

5.2 Cytarabine (AraC) 24

5.3 Study treatment overview 24

5.3.1 Treatment and observation 24
5.3.2 Phase I – part 25
5.3.3 Phase II – part 26
5.3.4 Dose modification and delays 26

5.4 Concomitant medications / therapy 28

6 STUDY PROCEDURES 29

6.1 Study evaluations 29
6.1.1 Evaluations before treatment 29
6.1.2 Evaluations during treatment 29
6.1.3 Evaluations in case of (suspected) relapse 30
6.1.4 Evaluations during observation of responding patients (CR, CRp, CRi) 30
6.1.5 Evaluations during follow-up in relapsed / refractory patients 30

6.2 Schedule of evaluations at baseline and during therapy 30

6.3 Central Bone marrow diagnostics and shipment modalities 32
6.3.1 Molecular genetic analyses 32
6.3.2 Requested material and shipment modalities 32
6.3.3 Cytomorphologic examination, Cytochemistry, Histology and Immunohistochemistry 33
6.3.4 Immunphenotyping 33
6.3.5 Central cytomorphology and immunophenotyping 33
6.3.6 Cytogenetics 34
6.3.7 Exploratory research 34

6.4 Duration of the study 35

6.5 End of study 35

6.6 Criteria for removal from study / premature end of study 35
6.6.1 Individual reasons (Criteria for removal of patients) 35
6.6.2 General reasons 35

7 ADVERSE EXPERIENCES 36

7.1 Definitions 36
7.1.1 Adverse Event (AE) 36
7.1.2 Adverse Reaction (AR) 37
7.1.3 Serious Adverse Event (SAE) or Serious Adverse Reaction (SAR) 37

7.2 Period of observation, documentation and reporting 39

7.3 Warnings and Precautions **41**
7.3.1 Investigational product (BIBF 1120) *41*
7.3.2 Cytarabine *43*

8 STUDY OUTCOME AND STATISTICAL ANALYSIS **44**

8.1 Power and Sample Size Calculation **44**

8.2 Statistical Analysis Plan **44**

8.3 Number of patients **44**

8.4 Efficacy **45**
8.4.1 Response criteria *45*

8.5 Definition of Study Endpoints **46**

9 QUALITY ASSURANCE **48**

9.1 Data Monitoring Committee **48**

9.2 Reference Laboratories **48**
9.2.1 Reference diagnostics for cytomorphology *48*

9.3 Monitoring **48**

9.4 Direct access to source data - documents **49**

9.5 Data Management **49**

10 INVESTIGATOR'S RESPONSIBILITIES, ETHICAL CONSIDERATIONS, CONFIDENTIALITY, PUBLICATION AND INSURANCE **50**

10.1 Investigator's responsibilities **50**
10.1.1 Declaration of Helsinki and GCP compliance *50*
10.1.2 Protocol adherence *50*
10.1.3 Documentation and retention of records *50*
10.1.4 Competent local authorities *51*

10.2 Ethical considerations **51**
10.2.1 Institutional Review Board / Independent Ethics Committee approval *51*
10.2.2 Informed Consent *51*

10.3 Confidentiality **52**

10.4 Publication **52**

10.5 Insurance **53**

11 REFERENCES ... 54

12 APPENDICES ... 56

12.1 Synopsis of the study (in German) ... 56

12.2 Performance Status ... 59

ATTACHMENTS

Attachment A	*Protocol agreement form*
Attachment B	*label of study medication*
Attachment C	*patient diary*
Attachment D	*patient information and informed consent form*
Attachment E	*cytarabine prescription information*
Attachment F	*clinical trial insurance certificate*
Attachment G	*clinical trial insurance terms & conditions*
Attachment H	*ethics committee approval*
Attachment I	*approval of competent authority (BfArm)*
Attachment J	*list of participating centers*

1 ABBREVIATIONS

AE	Adverse event
ALT	Alanine Transaminase
AML	Acute Myeloid Leukemia
AMLCG	AML Cooperative Group
AP	Alkaline phosphatase
APL	Acute promyelocytic leukaemia
AR	Adverse reaction
AraC	Cytosine arabinoside
AST	Aspartate Transaminase
ATIII	Antithrombin III
AUC	Area under Curve
bFGF	Basic Fibroblast Growth Factor
CBC	Complete Blood Count
CR	Complete Remission
CRc	Complete cytogenetic remission
CRi	Complete Remission with incomplete Neutrophil Recovery
CRm	Complete moleculargenetic remission
CRp	Complete Remission with incomplete Platelet Recovery
CRF	Case report form
CTCAE	Common Terminology Criteria for Adverse Events
CYP	Cytochrom P
DL	Dose Level
DLT	Dose Limiting Toxicity
DNA	Desoxyribonucleic acid
ECG	Electrocardiogram
ECOG	Eastern Cooperative Oncology Group
FAB	French-American-British cooperative group
FGF	Fibroblast Growth Factor
Flt	Fms like tyrosine kinase
FISH	Fluorescence in situ hybridization
GCP	Good Clinical Practice
G-CSF	Granulocyte Colony Stimulating Factor
GM-CSF	Granulocyte Macrophage Colony Stimulating Factor
IB	Investigator`s Brochure
IC50	Inhibitory Concentration with 50% Residual Enzyme Activity
ICH	International Conference on Harmonization
IRB/IEC	Institutional Review Board/ Independent Ethics Committee
ITD	Internal tandem duplication
LCK	Lymphocyte Specific Protein Kinase

LDH	Lactatedehydrogenase
MDS	Myelodysplastic Syndrome
MTD	Maximum Tolerated Dose
NPM	Nucleophosmin
ORR	Overall Response Rate
OS	Overall Survival
PB	Peripheral Blood
PDGF	Platelet Derived Growth Factor
P.O.	Peroral Administration
PTT	Partial Thromboplastin time
RAS	Rat sarcoma
RNA	Ribonucleic Acid
SAE	Serious Adverse Event
S.C.	Subcutaneous injection
SUSAR	Suspected Unexpected Serious Adverse Reaction
t_{max}	Time to maximal plasma concentration
UAR	Unexpected Adverse Reaction
ULN	Upper Limit of Normal
VEGF	Vascular Endothelial Growth Factor
WHO	World Health Organization
WWU	Westfälische Wilhelms Universität
ZKS	Zentrum für Klinische Studien Münster

2 BACKGROUND INFORMATION / INTRODUCTION

2.1 Condition background and current treatment

Acute Myeloid Leukemia (AML) is a clonal, malignant disorder that results from genetic and epigenetic changes in pluripotent stem or slightly more differentiated progenitor cells. The aberrant cells gain a growth and/or survival advantage in relationship to the normal pool of stem cells. Of patients over 60 years of age, only 25% survive two or more years after an intensive cytotoxic treatment [1]. In patients with medical contraindications against standard cytotoxic treatment, the therapy is considered palliative. Standard of care in patients with 20 – 30% bone marrow blasts unfit for an intensive treatment is 5-azacitidine, since Fenaux et al. demonstrated a survival benefit compared to conventional care [17]. In patients with >30% bone marrow blasts and non-adverse cytogenetic risk profile unfit for intensive treatment options, standard of care is low-dose cytarabine, since Burnett et al. could demonstrate a superiority over hydroxyurea with a complete response rate of 18% (compared to 1% with hydroxyurea) and a overall survival of 25% after one year [2]. However, patients with an adverse cytogenetic risk profile (i.e. complex-aberrant karyotype, aberrations including loss of chromosome 7q or 5q) do not respond to low-dose cytarabine [2] and no standard of care exists for those patients if not qualifying for an intensive treatment or treatment with 5-azacytidine.

Molecular pathogenesis

In the last decades, significant progress has been made about the molecular events inducing leukemic transformation in AML (reviewed in [3]). Although AML is a heterogeneous disease, common features of the leukemic blasts include a high proliferative potential, increased stem cell self renewal and a block in differentiation at a relatively immature state, within the mitotic pool. It is now widely accepted that AML blasts already harbor several transforming events when the disease becomes clinically apparent. Many AML-specific oncogenic mutations and epigenetic alterations have been identified and their ability to cause leukemia has been analyzed in primary and human xenograft mouse models. A model has been suggested that at least two mutations from different complementation classes have to accumulate in a myeloid progenitor cell to cause AML [4]. One of these mutations is thought to cause deregulation of transcriptional programs needed for the orchestration of myeloid differentiation. Thus, more than 50% of all AML cases have been shown to contain a mutation in a transcriptional regulator, often as a result from balanced reciprocal translocations.

These mutations often coincide with mutations in signal transduction mediators. Again, about 50% of AML cases have been shown to contain a mutation in a signaling mediator, most frequently in receptor tyrosine kinases or in the Ras oncogene. The receptor tyrosine kinase Flt3 has been found to be the

most common target among these, in 30% of all AML cases and a lower incidence in elderly patients [5]. Flt3 mutations have been shown in several *in vitro* and animal models to cause malignant transformation of myeloid cells and to cooperate with other known oncogenes to cause an AML-type disease in mice.

The more common type of Flt3-mutations, so-called internal tandem duplications, are associated with a bad prognosis of AML patients, especially when the normal Flt3 allele is lost in the majority of blasts [6]. Flt3 mutations are very frequently associated with mutations in the gene for nucleophosmin (NPM), a protein with functions in organizing the traffic of proteins between the nucleus and the cytoplasm. Other very common mutations in a signal transduction mediator are oncogenic N- and K-Ras mutations that have been described in AML for over 20 years, with a prevalence of slightly less than 30% [7]. The functional consequences of these mutations are not yet known.

The role of increased angiogenesis in AML has been elucidated since the beginning of this century [8, 9]. Bone marrow infiltrated by AML blasts exhibits an increased microvessel density compared to normal bone marrow and microvessel density decreased in response to induction chemotherapy [9]. Hypothetically, growth factors expressed by AML blasts stimulate the growth of vascular endothelial cells and vice versa. This hypothesis is supported by findings that the proangiogenic growth factor vascular endothelial growth factor (VEGF) as well as the VEGF receptors 1 (VEGFR1 / Flt-1) and 2 (VEGFR2 / KDR) are expressed in AML cells, and human endothelial cells exhibit an increase in the expression of the myelogenous growth factor granulocyte-macrophage colony stimulating factor (GM-CSF) upon stimulation with VEGF [10]. Several data suggest that a stimulating effect of VEGF signalling on AML blasts is mediated by VEGFR2: VEGFR2, but not VEGFR1, is overexpressed in human untreated AML bone marrow samples and the expression correlates with the bone marrow microvessel density [11]. Upon induction of a CR, VEGFR2 levels decreased to normal range. Another receptor tyrosine kinase mediating proangiogenic effects is the basic fibroblast growth factor (bFGF). In AML, an overexpression of bFGF in bone marrow and an autokrine stimulation of AML blasts upon incubation with bFGF could be observed [12]. Furthermore, stromal secretion of PDGF has been reported as being important also in the biology and progression of AML [13].

In summary, AML is thought to be the result of multiple events mediating a block of differentiation, inhibition of apoptosis, enhanced bone marrow angiogenesis and enhanced stem cell self renewal and the combined inhibition of VEGF-, bFGF- and PDGF-signalling might be a promising target for AML therapy.

2.2 Investigational product background

BIBF 1120 is a potent, orally available triple kinase inhibitor targeting VEGFRs, PDGFRs, and FGFRs. The specific and simultaneous abrogation of these pathways results in effective growth inhibition of both endothelial and, via PDGF- and FGF-receptors of perivascular cells, which may be

more effective than inhibition of endothelial cell growth via the VEGF pathway alone. BIBF displays growth inhibitory and proapoptotic effects in various AML cell lines in the nanomolar range [14].

Phase I dose selection studies revealed that BIBF 1120 is generally well tolerated with mild to moderate adverse effects such as gastrointestinal symptoms (nausea, diarrhea, vomiting, abdominal pain) and reversible elevations of liver enzymes. Initial signs of clinical activity including an encouraging rate of patients with stabilization of their tumor of 54% and 68%, respectively, have been observed in patients with various solid tumors.

2.2.1 Preclinical information

2.2.1.1 General information

BIBF 1120 is a potent small molecule triple receptor tyrosine kinase inhibitor (PDGFR α/β [platelet derived growth factor receptor], FGFR 1/3 [fibroblast growth factor receptor], VEGFR 1-3 [vascular endothelial growth factor receptor]). VEGFR-2 is considered to be the crucial receptor involved in initiation of the formation as well as the maintenance of tumor vasculature. On the molecular level, BIBF 1120 is thought to inhibit the signalling cascade mediating angiogenesis by binding to the adenosine triphosphate (ATP) binding pocket of the receptor kinase domain, thus interfering with cross-activation via autophosphorylation of the receptor homodimers. Besides inhibition of neo-angiogenesis, tumour regression may also be achieved by inducing apoptosis of tumour blood vessel endothelial cells. Inhibition of receptor kinases may also interfere with autocrine and paracrine stimulation of tumour angiogenesis via activation loops involving VEGF, PDGF, and bFGF utilized by perivascular cells such as pericytes and vascular smooth muscle cells. In vitro, the target receptors are all inhibited by BIBF 1120 in low nanomolar concentrations. In in vivo nude mouse models, BIBF 1120 showed good anti-tumour efficacy at doses of 50 – 100 mg/kg, leading to a substantial delay of tumour growth or even complete tumour stasis in xenografts of a broad range of differing human tumour types. Histological examination of treated tumours showed a marked reduction of tumour vessel density by approximately 80%.

2.2.1.2 In vitro efficacy

The in vitro potency of BIBF 1120 as a VEGFR-2 kinase inhibitor has been determined in enzymatic assays using the human VEGFR-2 kinase protein domain and in cellular assays measuring the inhibition of VEGF-stimulated human umbilical cord vein endothelial cell (HUVEC) proliferation. VEGFR-2 kinase assays revealed an IC50 of 21 nM. The VEGFR-3 kinase was inhibited with an IC50 of 13 nM and for FGFR-1 (IC50=69 nM), FGFR-3 (IC50=137 nM) and PDGFRα/β (IC50=59 nM/60 nM) a slightly lower potency was detected. These receptors are expressed on perivascular cells, such as pericytes and smooth muscle cells, that are also involved in tumour angiogenesis and therefore their inhibition may contribute to the overall efficacy of BIBF 1120. More than 20 other kinases were also

analysed and showed no inhibition by the compound (e.g. insulin receptor, HER2, and several cyclin dependent kinases) with the exception of the three members of the Src family of tyrosine kinases: Src (IC50=156 nM), lymphocyte specific protein kinase (lck) (IC50=16 nM), and lyn (IC50=195 nM). Recent data suggest that src family kinases exhibit antiapoptotic effects on AML blasts mediated by phosphorylation of STAT5 [15]. The inhibition of VEGF-stimulated HUVEC and human skin microvessel endothelial cells (HSMEC) proliferation showed an IC50 of 9 nM and 12 nM, respectively. The proliferation of pericascular cells such as pericytes and vascular smooth muscle cells (both stimulated with PDGF-BB) was inhibited with IC50 values of 76 nM and 55nM, respectively. In myeloid cell lines, the sensitivity was heterogenous with IC50 values between 8 nM (Kasumi-1) and > 1,000 nM [14].

2.2.1.3 Pre-clinical pharmacokinetics

Intravenous administration:

After iv administration plasma clearance was high in the rat (~ 200 mL/[min·kg]) and moderate to high in both monkey species (~ 30 to 40 mL/[min·kg]). Half-lives of about 4, 6 and 7 h were calculated for rat, Cynomolgus and Rhesus monkey, respectively. The high volume of distribution (Vss) of about 41, 9 and 10 L/kg indicated a good tissue penetration of the compound. There was no evidence for a gender effect, the exposure to BIBF 1120, BIBF 1202 and BIBF 1202-glucuronide increased almost proportionally with the dose in the rat.

Oral administration:

In the rat, no influence of the suppression of gastric acid secretion on oral absorption was observed, indicating that normal acid secretion is not essential for absorption of BIBF 1120 given as the final salt form. Furthermore the formulation as solution or suspension in lipid matrix had no impact on the bioavailability. The absolute bioavailability was about 12 % in the rat. The pharmacokinetic characteristics were very similar between Cynomolgus and Rhesus monkey. C_{max}, t_{max}, and AUC0-24 increased proportionally with the dose up to an oral dose of 80 mg/kg. The absolute bioavailability was about 15 to 20% in both monkey species. Exposure was assessed in toxicity studies in Wistar rats and Cynomolgus and Rhesus monkeys. Across studies, the variability was assessed as moderate. Exposure increased proportionally with the dose in the monkey but slightly more than proportionally in the rat. No accumulation and no difference in gender were observed

2.2.1.4 Pre-clinical toxicology

Data from four-week, 13-week, and 26-week toxicity studies in rats as well as four-week, 13 week and 52-week toxicity studies in monkeys are available. Relevant histopathological findings in these studies were observed in the gastrointestinal tract, lymphatic tissues, kidneys, bone marrow, liver, extrahepatic bile duct, exocrine glands, and the skin. Bone changes in growing animals (thickening of epiphyseal growth plate) were interpreted as a typical mechanism-related toxicity associated with a VEGFR-2

inhibitor. Mild changes in hematological and clinical chemistry parameters (increases in γ-glutamyl transferase [γ-GT], aldolase, alanine amino transferase [ALT], aspartate aminotransferase [AST], leucine aminopeptidase [LAP], glutamate dehydrogenase [GLDH]) were seen in rats. Minimal to slight changes in immunotoxicological parameters (CD4 count) and lymphoid tissues may be the correlate to the additional inhibition of src family non-receptor tyrosine kinases such as lck and lyn. Overall, the histopathological findings and changes of laboratory parameters were mild to moderate and generally confined to the high dose groups.

BIBF 1120 is non-mutagenic, even at high doses. One compound in a batch of potential degradation products that may be formed under systemic and/or acidic conditions was found to be weakly Ames positive at high concentration after metabolic activation, while a second batch of the same products was Ames negative. The compound was not found in any of the drug substance batches of BIBF 1120 used and thus did not occur within the limits of detection. Further experiments (mouse lymphoma assay, micronucleus assay) indicated that the compound does not raise a safety concern for cancer patients.

Two exploratory studies in rats revealed a teratogenic effect of BIBF 1120 with a steep dose/effect relationship and an early onset of embryofetal deaths at low dosages. This effect was observed at dose levels resulting in plasma drug concentrations comparable to or below those in humans. Because the concentration of BIBF 1120 in semen is unknown, males receiving BIBF 1120 and having sexual intercourse with females of childbearing potential should use latex condoms.

2.2.2 Clinical information

As of July 10, 2009, a total of 739 cancer patients, 423 patients with idiopathic pulmonary fibrosis and 59 healthy volunteers have been treated in multiple dose studies with BIBF 1120 or blinded BIBF 1120 / Placebo [16]. The predominant adverse events were nausea, diarrhoea, vomiting, abdominal pain and fatigue of mostly low to moderate intensity after monotherapy with BIBF 1120. Dose limiting toxicities were dose dependent hepatic enzyme elevations that were reversible after discontinuation of BIBF 1120 treatment.

These liver enzyme elevations were only in few cases accompanied by a simultaneous increase of bilirubin. In general common terminology criteria for adverse events (CTCAE, version 3.0) grade 3 liver enzyme increases were reported in the dose groups of 250 mg twice daily or higher. They also were reversible and usually occurred within the first two months of treatment.

Combination of BIBF 1120 with other anti-cancer drugs revealed a similar adverse event profile as compared to BIBF 1120 monotherapy except for the chemotherapy related toxicities. There was no change of the pharmacokinetic parameters of BIBF 1120 or of the cytotoxic compounds due to the combined treatment. Dose limiting toxicity consisted mostly of liver transaminase elevations as in the

monotherapy phase I trials with the exception of the combination of BIBF 1120 with pemetrexed, where fatigue was the most relevant dose limiting toxicity.

Hypertension or thromboembolic events were rare and did not suggest an increased frequency as a consequence of therapy with BIBF 1120.

All adverse events observed in healthy volunteers after single administration of BIBF 1120 were of CTCAE Grade 1 intensity and fully reversible.

Available pharmacokinetic data indicate that the systemic exposure required for biological activity can be achieved in cancer patients. Maximum plasma concentrations occurred mainly 1 to 4 hours after administration. There was no deviation from a dose proportionality in the pharmacokinetic of BIBF 1120 detectable. Steady state was latest reached within 9 days of treatment. The gMean terminal half-life was between 7 to 19 hours. The main metabolite of BIBF 1120 was BIBF 1202 which was in vitro further glucuronidated to the BIBF 1202 glucuronide via the UDP glucuronosyltransferase (UGT) 1A1 enzyme. In humans, 93.4% of total [14C] radioactivity was excreted in the faeces within 120 hours after oral administration of BIBF 1120. Only 0.7 % of total [14C] radioactivity was eliminated via the urine.

In the phase I trials where BIBF 1120 was combined with chemotherapeutic regimens, there was no change of the pharmacokinetic parameters of BIBF 1120 or of the cytotoxic compounds due to the combined treatment.

Data from patients with advanced solid tumors demonstrate a pharmacodynamic effect of BIBF 1120 treatment on selected tumor lesions as shown by dynamic contrast-enhanced magnetic resonance imaging (DCE-MRI). Based on pharmacokinetic analysis, a sufficient systemic exposure for biologic activity was observed in these advanced cancer patients.

Signs of efficacy, comparable to historical data of other VEGFR inhibitors, were reported in patients with advanced non small cell lung cancer when BIBF 1120 was administered as monotherapy. Signs of efficacy were also observed in the Phase I combination trials with other anti-cancer drugs including complete and partial responses.

Based on the Phase I dose escalation trials with BIBF 1120 monotherapy, the maximum tolerated dose was defined to be 250 mg for twice daily dosing in Caucasians and 200 mg twice daily in Japanese patients with a manageable safety profile in advanced cancer patients. The maximum tolerated dose for combination therapy of BIBF 1120 and other anti-cancer drugs (such as docetaxel, paclitaxel, pemetrexed, carboplatin, 5-FU, oxaliplation) was determined to be 200 mg twice daily. Based on the overall safety profile a dose of 200 mg twice daily of BIBF 1120 is the recommended phase III dose for combination treatments with pemetrexed, docetaxel and paclitaxel/carboplatin. Available pharmacokinetic data indicate that the systemic exposure needed for biological activity can be achieved starting with doses of 100 mg BIBF 1120 once daily.

2.2.2.1 Combination with chemotherapeutic agents

101 patients have been treated in six phase I combination trials investigating the combination of BIBF 1120 with docetaxel, pemetrexed, paclitaxel/carboplatin and FOLFOX6 and phase III placebo-controlled, double-blind studies combining BIBF 1120 or placebo with docetaxel, pemetrexed are ongoing.

The pattern of drug related AEs observed in trials combining BIBF 1120 with cytotoxic agents was similar to the adverse event profile of the phase I monotherapy trials. The most common gastrointestinal AE's were nausea, vomiting, diarrhoea and abdominal pain of mostly low to moderate intensity. Asthenia or fatigue was reported more frequently compared to the phase I monotherapy trials. Dose limiting toxicity consisted mostly of liver transaminase elevations as in the monotherapy phase I trials, with the exception of the 1199.18 trial of BIBF 1120 in combination with pemetrexed, where fatigue was the most relevant dose limiting toxicity (DLT). Grade 3 transaminase increases mostly occurred at doses of 250 mg bid and rarely at 200 mg. bid. With one exception (a patient who also suffered from HCV infection), no grade 3 transaminase elevation occurred at doses below 200 mg bid. Adverse events such as alopecia, haematotoxicity and skin toxicity which are known side effects of the selected combination partners occurred more frequently in the phase I combination trials compared to the monotherapy phase I studies. There was no discernible dose dependency for these side effects according to the dose of BIBF 1120. As in the phase I monotherapy trials, hypertension or thromboembolic events were not frequent and did not suggest an increased frequency during therapy with BIBF 1120. The recommended dose of BIBF 1120 was 200 mg bid in combination with all combinations tested. In all trials, data suggested that addition of BIBF 1120 to standard cytotoxic regimens may have the potential to enhance clinical benefit of the standard treatment regimens.

2.2.2.2 Clinical pharmacokinetics

After oral administration, BIBF 1120 maximum plasma concentrations (t_{max} 1 to 4 h) and AUC increased dose-proportionally after single administration as well as at steady state following single daily and bidaily dosing. Steady state was reached latest within 9 days. After food intake, a trend towards an increased systemic exposure (around 20%) which was not statistically significant, and a delayed absorption was observed compared to administration of BIBF 1120 under fasted conditions. A high apparent volume of distribution during the terminal phase and a high apparent total plasma clearance for BIBF 1120 was observed. The terminal half-life varied between 7 and 19 h. The major route of elimination of total [^{14}C] radioactivity was via biliary excretion (93.4%). The contribution of renal excretion was low (0.7%). Pharmacokinetic behavior of BIBF 1120 is similar between healthy volunteers and cancer patients, and in patients with different cancer types. As expected, there was no clinically relevant drug-drug interaction between BIBF 1120 and paclitaxel, docetaxel, carboplatin or pemetrexed at clinically used doses. All principle metabolites that were observed in humans have been found in animal studies (rats and Rhesus monkeys) after oral dosing. The principal (major)

metabolites, both in humans and animals, are BIBF 1202 and its glucuronide. Plasma concentrations needed for biological activity at VEGFR 1, 2 and 3 (compared to in vitro inhibitory concentrations) can be achieved starting with doses of 100 mg of BIBF 1120 once daily.

3 STUDY OBJECTIVES

3.1 Rationale for this study

Despite recent advances into the insight of the molecular mechanism of acute myelogenous leukemia, not much improvement in the still unsatisfactory treatment of patients > 60 years has been made with standard therapy of cytarabine and antracyclines, followed by intermediate-dose cytarabine consolidation. For patients medically unfit for this standard chemotherapy, the current standard of care consists of 5-azacytidine in patients with <30% bone marrow blasts, low-dose cytarabine in patients with non-adverse cytogenetic risk profile and >30% bone marrow blasts and best supportive care only with the addition of cytoreductive therapy, i.e. hyxdroxyurea, if required for leukocytosis, in patients with adverse cytogenetics and >30% bone marrow blasts. Despite different potential therapeutic options, outcome in this patient population is still dismal.

A body of data suggests that AML blasts are dependent on signal transduction pathways to mediate enhanced proliferation, apoptosis inhibition and block of differentiation. In addition, safety data suggest a good safety profile of BIBF 1120, compared to intensive standard induction therapy. For this reasons, the addition of the triple kinase inhibitor BIBF 1120 to low-dose cytarabine might improve therapy results in elderly AML patients unfit for intensive induction therapy.

3.1.1 Benefit / risk ratio

Patients eligible for this trial have a chance of survival with any other therapy of 25% after one year [2]. The risk of this trial is that addition of BIBF 1120 to low-dose cytarabine increases treatment-related death due to extensive toxicity. Since a MTD for BIBF 1120 monotherapy and for combination therapy with various cytotoxic agent has already been evaluated in clinical trials in solid tumors, it seems justified to perform an enhance dose finding for the combination therapy of BIBF 1120 and low-dose cytarabine.

The possible benefit of the trial is to decrease the rate of treatment failure in the trial population. For a single patient, this could result in increased life-span, ideally without evidence of leukemia. Given the extensively high rate of primary or secondary treatment failure in this patient population, the medical need for novel, rational therapeutic approaches for this patient population is overwhelming. As pointed out above, the molecular rationale for the therapeutic administration of BIBF 1120 in AML is well founded. Thus, the benefit/risk ratio for a patient to participate in this trial appears favorable.

Since the application of 5-azacytidine has a proven efficacy in low-proliferating AML with ≤ 30% bone marrow blasts [17], AML patients with ≤ 30% bone marrow blasts should be treated with 5-azacytidine if possible. Accordingly, patients qualifying for an intensive treatment have a chance of achieving a complete remission of 50% and should therefore treated with an intensive approach.

However, no therapeutic approach exists in patients with >30% bone marrow blasts and adverse cytogenetic risk unfit for an intensive treatment, and these patients have the highest need for therapy improvement. Patients with non-adverse cytogenetic risk and not qualifying for both therapy with 5-azacytidine and intensive chemotherapy only have an 18% chance of achieving a complete remission upon treatment with low-dose cytarabine alone. It therefore seems justifiable to include these patients (with AML not qualifying for both 5-azacytidine and intensive chemotherapy) into this trial.

3.2 Study objectives

3.2.1 Phase I

Primary endpoint:

- to evaluate the safety and tolerability of BIBF 1120 combined with low-dose cytarabine

3.2.2 Phase II

Primary endpoint:

- to evaluate the overall response rate (ORR, consisting of: CR, CRp and CRi as defined by IWG criteria[1]) of the whole study population

Secondary endpoints:

- to compare the ORR rate of the Flt3-mutated patients versus the Flt3-wildtype patients and within different cytogenetic risk groups
- to evaluate the CR rate of the whole study population
- to compare the CR rate of the Flt3-mutated patients versus the Flt3-wildtype patients and within different cytogenetic risk groups
- to evaluate the one-year overall survival (OS) of the whole study population
- to compare the one-year overall survival (OS) of the Flt3-mutated patients versus the Flt3-wildtype patients and within different cytogenetic risk groups
- to evaluate the relapse-free survival of the responding patients
- to evaluate the time to response (CR, CRp or CRi) of the responding patients
- to evaluate the toxicity in the whole study population
- to evaluate the development of biomarkers indicating the course of disease, including genetic, epigenetic, transcriptional, protein and phosphoprotein markers in leukemic blasts, bone marrow, peripheral blood cells, serum and plasma, and bone marrow microvessel density.

[1] response criteria by the International Working Group (IWG) are defined as:

- Complete remission (CR): less than 5% bone marrow blasts and regeneration of both peripheral neutrophils to >1,000/µl and platelets to >100,000/µl
- Complete remission with incomplete platelet recovery (CRp): less than 5% bone marrow blasts and regeneration of peripheral neutrophils to >1,000/µl with a platelet count of <100,000/µl
- Complete remission with incomplete neutrophil recovery (CRi): less than 5% bone marrow blasts with a neutrophil count of <1,000/µl with or without platelet recovery

3.3 Study design

This is a single-arm, prospective, multi-center phase I/II study. Patients 60 years or older with relapsed or refractory (phase I only) or newly diagnosed AML medically unfit for or unwilling to receive an intensive standard chemotherapy will receive intermittent low-dose cytarabine s.c. combined with a continuous treatment with BIBF 1120. Dose escalation in the phase I part will be a classical 3 + 3 design with an increased starting dose level due to the experiences of the MTD determination in monotherapy studies. For further details see chapter 5.

4 SELECTION, REGISTRATION AND WITHDRAWAL OF PATIENTS

4.1 Number of patients

According to the power calculations given in 8.1, n = 122 + X patients will be included in the phase II of the study.

4.2 Admission criteria

4.2.1 Inclusion criteria

- Patients with newly diagnosed AML (except APL) according to the FAB or WHO classification, including AML evolving from MDS or other hematological diseases and AML after previous cytotoxic therapy or radiation (secondary AML), with medical contraindications against or not willing to receive a standard induction and consolidation therapy.
- Bone marrow aspirate or biopsy must contain > 20% blasts of all nucleated cells. In AML FAB M6 ≥ 30% of non-erythroid cells in the bone marrow must be leukemic blasts. In patients with 20-30% blasts, the indication for a treatment with hypomethylating agents (5-azacitidin or decitabin) should be considered prior to inclusion into the trial.
- Age ≥ 60 years
- Informed consent, personally signed and dated to participate in the study
- Male patients enrolled in this trial must use adequate barrier birth control measures during the course of treatment and for at least 3 months after the last administration of study therapy (low-dose cytarabine and/or BIBF 1120).

4.2.2 Exclusion criteria

- Patients with 20-30% bone marrow blasts which are qualifying for and consenting into a therapy with hypomethylating agents
- Patients who are eligible for and consenting into a standard chemotherapy
- Known central nervous system manifestation of AML
- Inadequate liver function (ALT and AST ≥ 2.5 x ULN) if not caused by leukemic infiltration
- Known chronically active hepatitis C infection or acute hepatitis

- Chronically impaired renal function (creatinin clearance < 30 ml/min)
- Uncontrolled hypertension with a resting pressure systolic > 160 mmHg or diastolic > 95 mmHg despite adequate treatment
- severe trauma or surgery within 4 weeks of study entry
- severe, non-healing wounds, ulcer or fracture
- Uncontrolled active infection
- Concurrent malignancies other than AML or other severe diseases which in the opinion of the investigator are likely to influence the endpoint asessment
- Hypersensitivity to cytarabine (not including drug fever or exanthema)
- Phase II only: Previous treatment of AML except hydoxyurea up to 24 hours before study medication
- Phase II only: Previous therapy with tyrosine kinase inhibitors or angiogenesis inhibitors
- Parallel participation in another clinical trial for the same indication. Eligibility of patients with investigational drug therapy outside of this trial during or within 4 weeks of study entry should be discussed with the study office prior to study entry
- Any severe concomitant condition, which makes it undesirable for the patient to participate in the study or which could jeopardize compliance with the protocol

4.3 Patient registration

4.3.1 Registration

Registration will be performed by the Department of Medicine A in Muenster during regular office hours (Monday through Friday 8 am to 5 pm) either by phone: 0251 / 83-49963 or per fax: 0251 / 83-47599.

Details for registration are:

- sex and date of birth
- Diagnosis (according to the FAB-classification)
- Study centre and name of the physician

During the registration process, a patient registration number will be assigned to the patient by the IBCR with the following format:

ZZZ – NNN

ZZZ = three numbers for the Center

NNN = Unique patient identification number (continuously increasing number)

4.4 Withdrawal of patients

A patient is free to withdraw from the study at any time for any reason without prejudice to his/her future medical care by the physician or at the institution. The Investigator may also withdraw the patient at any time in the interest of patient safety. The primary reason for withdrawal must be recorded in the patient's medical record and on the withdrawal form in the Case Report Form (CRF).

5 STUDY, STANDARD AND CONCOMITANT TREATMENTS

5.1 Investigational product

BIBF 1120 is a small molecule receptor tyrosine kinase (RTK) inhibitor, with competitive inhibition of ATP-binding to the ATP-binding site. In dosages above 100 mg once daily, it inhibits the activity of the RTK VEGFR 1-3, FGFR1, FGFR3, PDGFRα and PDGFRβ. For full details of the pre-clinical and clinical information please refer to the summary of product characteristics and the investigators brochure.

5.1.1 Relevant physical, chemical and pharmaceutical properties

BIBF 1120 is a yellow, highly crystalline, hemihydrous powder with a high water solubility. BIBF 1120 is provided as soft gelatin capsules containing a suspension of milled active as the salt. It is available in dose strengths corresponding to 100 mg (peach, oblong capsules) and 150 mg (brown, oblong capsules).and is packaged in bottles containing 30 capsules.

5.1.2 Instructions for storage and handling

In the solid state BIBF 1120 is stable with a shelf life of 36 months. The capsules are packaged in bottles and have to be stored below 30°C. BIBF 1120 will be swallowed unchewed after a meal every 12 hours (± 1 hour), i.e. daily at about the same time in the morning and in the evening.

5.1.3 Interactions with concomitant medications

In vitro data showed that BIBF 1120 has no potential for drug-drug interactions via CYP-450-enzymes.

5.1.4 Drug accountability

The drug will be distributed to the responsible investigator in the center in vials containing the drug supply sufficient for two therapy cycles (4 x 30 capsules for dose levels 1 [100 mg bidaily] and 2 [150 mg bidaily], 8 x 30 capsules for dose level 3 [200 mg bidaily]). Should a daily dose of 2 x 200 mg be evaluated as the recommended dose, an additional vial of 150 mg capsules (30 capsules) will also distributed to the centers by the initial drug distribution to cover dose reductions. For each dose level within the phase I part and for the phase II part, each center will be supplied with an initial delivery of study medication sufficient for two cycles.

The center is responsible for ordering more supply at least three working days ahead of time.

Each center has to document in a list, when which drug was given to whom. This list has to contain the date, the patients name, the batch number, the use-by date and the number of vials dispensed.

The patient or the person applying the drug has to document the drug application and - in case of a failure to take the medication - a short statement, why medication was not taken, in a standardized

diary provided by the study center. Leftover study medication will be collected by the study center upon regular visits.

5.2 Cytarabine (AraC)

Since the low-dose cytarabine treatment used in this protocol is approved and belongs to the standard of care of the population included in this study, the low-dose cytarabine treatment is NOT an investigational product in this study.

Cytarabine belongs to a group of chemotherapeutic agents called antimetabolites. Although the mechanism of action is not completely understood, it appears that cytarabine acts through the inhibition of DNA polymerase. A limited, but significant, incorporation of cytarabine into both DNA and RNA has also been reported.

Cytarabine is not active orally. It may be given by intravenous infusion or injection, subcutaneously, or into the liquor space. When large intravenous doses are given quickly, patients are frequently nauseated and may vomit for several hours after injection. This problem tends to be less severe when the drug is infused.

A cytarabine syndrome has been reported to occur in patients who received cytarabine. It is characterized by fever, myalgia, bone pain, occasionally chest pain, maculopapular rash, conjunctivitis and malaise. It usually occurs 6-12 hours following drug administration. Corticosteroids have been shown to be beneficial in treating or preventing this syndrome. If the symptoms of the syndrome are deemed treatable, corticosteroids should be contemplated as well as continuation of therapy with cytarabine.

Like other cytotoxic drugs, cytarabine may induce anemia, leukopenia, thrombocytopenia and hyperuricemia secondary to rapid lysis of neoplastic cells.

Rarely, severe skin rash, leading to desquamation has been reported.

For full details of the drug information please refer to the “Fachinformationsverzeichnis Deutschland” in its latest version.

5.3 Study treatment overview

5.3.1 Treatment and observation

Patients will receive low-dose cytarabine in a dose of 2 x 20 mg as subcutaneous injection twice daily on days 1-10 of each cycle and BIBF 1120 twice daily (for dose level, see below) as an oral dose throughout the cycle on a 28-day cycle. A bone marrow aspirate (core biopsy in case of a dry tab) and a complete blood count (CBC) with differential blood count for response evaluation will be performed between days 24 and 28 of each cycle. Patients will receive additional cycles from day 29 on for up to six cycles in total if the following applies: I. absence of significant disease progression as judged by

the investigator, II. absence of unacceptable toxicity, and III. no adequate response (CR, CRp or CRi) in the bone marrow evaluation between days 24 and 28. If either the application of cytarabine on day 29 is undesirable in the opinion of the investigator, or if I. and II. applies and the response evaluation is pending on day 29, the application of the next cycle can be postponed until recovery of the patient and / or result of the bone marrow evaluation. In this case, patients will be bridged to the next cycle with BIBF 1120 at the last dosage of the previous cycle. In case of the achievement of a CR, CRp or CRi, patients switch to observation and in case of progressive disease or intolerable toxicity patients will be observed for survival (see 5.3.2 and 5.3.3).

Treatment Plan:

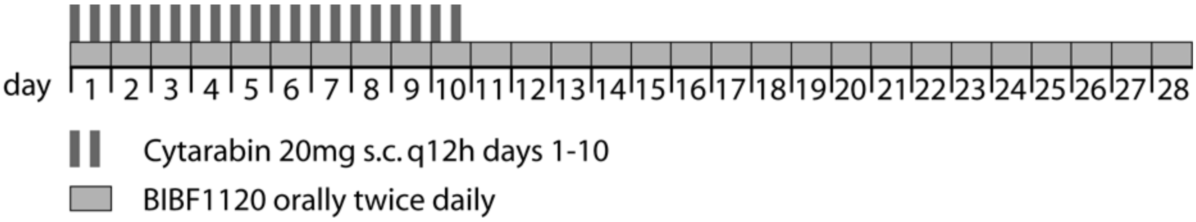

Treatment overview:

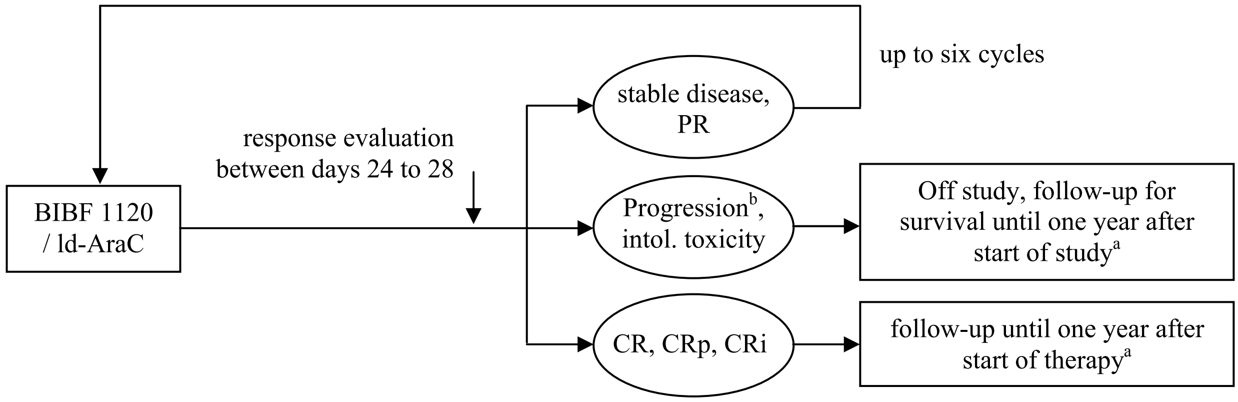

[a] In phase I, patients will be followed up only until 28 days after the last administration of study medication..

[b] Clinically meaningful disease progression as assessed by the investigator. Assessment takes into account, but is not restricted to: blast count in bone marrow and peripheral blood, tumor lysis parameters, extramedullary manifestations, and worsening of clinical or laboratory parameters or general condition attributable to leukemia progression.

5.3.2 Phase I – part

The optimum dosage of BIBF 1120 in combination with low-dose cytarabine will be determined in a phase I part. Based on acceptable safety data for BIBF 1120 monotherapy, there will be a rapid dose

finding. Dose finding will proceed in a classical 3+3-design. Predefined dose levels are: 100mg (dose level 1), 150mg, (dose level 2), and 200mg (dose level 3) twice daily. A cohort of three patients will be treated in each dose level and toxicity will be assessed in the first treatment cycle (28 days) by Common Terminology Criteria for Adverse Events V 3.0 (CTCAE). In case of no dose limiting toxicity (DLT), the dose will be increased by one dose level. In case of one DLT, three further patients will be recruited into the same dose level. If >1 DLT occurs in three or six patients, the next lower dose level will be defined as the maximum tolerable dose (MTD) and will be evaluated in the phase II part of the study after approval of the data monitoring committee (DMC). In case of >1 DLT in the dose level 1 cohort, the dose will be decreased to dose level 0 (100 mg BIBF 1120 once daily). The study will not proceed to the phase II part if >1 DLT occurs in dose level 0. A DLT is defined as every severe adverse reaction CTC grade IV with possible or definite relationship to the study drug. SAE's due to leukemia progression (i.e. pancytopenia, anemia, leukopenia / neutropenia, thrombocytopenia, neutropenic fever, neutropenic infections, thrombocytopenic bleedings) are excluded from DLT's. Patients responding after the therapy (CR, CRp, CRi) or with progressive disease or intolerable toxicity will be observed until 28 days after the last application of study medication.

5.3.3 Phase II – part

In the phase II part, patients will receive the treatment as described in 5.3.1 with the dose level of BIBF 1120 as defined in the phase I part of the study. Prior to the start of the phase II, the dose level as determined in the phase I part will be amended to the protocol. Responding patients after the therapy (CR, CRp, CRi) will be observed until one year after therapy initiation. During the observation period of responding patients, monthly visits with CBC with differential blood count and a bone marrow aspiration every 3 months and additionally in case of suspected relapse will be performed (for details, see 6.1). In case of progressive disease or intolerable toxicity, study participation will be terminated and the patient will be observed for survival until one year after start of therapy. However, timelines for AE and SAE reporting apply during the observation period (see 7.2).

5.3.4 Dose modification and delays

The dose modifications of BIBF 1120 will follow the following pre-defined dose levels:

Dose level 3:	200 mg twice daily
Dose level 2:	150 mg twice daily
Dose level 1:	100 mg twice daily
Dose level 0:	100 mg once daily

5.3.4.1 General dose modifications

If a dose reduction below 100 mg once daily is required, the patient should be discontinued from the study treatment. After resolution of the adverse event the dose may be re-escalated at the discretion of the investigator. As a general rule, grade 3 toxicity should be followed by permanent dose reduction of BIBF 1120. Resolution of an adverse event is defined as disappearence or reduction of the adverse event to < grade 3 toxicity. For patients with grade 2 or greater toxicities present at baseline resolution to at least baseline levels will apply. No dose reductions / dose delays are required for non-hematological toxicities which are controlled with an effective specific treatment (i.e. Nausea / Emesis).

5.3.4.2 Dose modifications for hematological toxicity

For an effective antileukemic activity, a hematotoxicity is an unevitable side effect of the combination therapy. Since only patients with persisting leukemia receive additional chemotherapy / BIBF cycles, no routine dose modifications or dose delays are considered for hematological toxicity during the combination therapy. This also applies for complications of hematotoxicity like bleeding complications or neutropenic infections. If, however, a continuation of the combination treatment places the patient to an inadequate risk in the opinion of the investigator, study treatment including treatment with cytarabine should be discontinued.

5.3.4.3 Dose modifications for non-hematological toxicity

The general recommendations for non-hematological toxicities are listed in table 1-2.

Table 1-2: Non-hematological Criteria for Dose Delay and Dose Modification of BIBF 1120		
Grade	Dose Delay	Dose Modification
Grade 0-2	Treat on time	No Change
Grade 3	DELAY [a] until ≤ Grade 2	DECREASE one dose level [b]
Grade 4	OFF protocol therapy	OFF protocol therapy

Table 1-2: Non-hematological Criteria for Dose Delay and Dose Modification of BIBF 1120
a. If no recovery after 30 days delay, treatment will be discontinued<br>b. If a dose reduction below 100 mg once daily is required, treatment will be discontinued

These recommendations pertain, if the adverse event is considered to be related to BIBF 1120, but not to cytarabine.

5.4 Concomitant medications / therapy

All appropriate palliative and supportive care for disease-related symptoms will be provided to all patients. Symptomatic treatments should be used as needed and may be given as prophylactics in subsequent cycles.

Anti-microbial prophylaxis in the presence of neutropenia should be given according to the study centers' standard procedures.

It is recommended that the use of G-CSF is restricted to therapeutic use in severe infections and to patients with CRi. However, the use of C-GSF or other growth factors may be expanded to additional selected cases at the discretion of the investigator. It is also to the discretion of the participating centers, which type of G-CSF preparation they choose to treat their patients.

Hydroxyurea and / or leukapheresis may be used according to the center`s standard practice to decrease initial hyperleukocytosis (leukocytes ≥ 100,000/µl or symptoms of leukostasis) before starting the first therapy cycle.

6 STUDY PROCEDURES

6.1 Study evaluations

Most procedures for patient evaluation that are listed below are considered by the investigators to be necessary according to the standard of good clinical practice for the diagnosis and treatment monitoring of AML patients.

6.1.1 Evaluations before treatment

- Complete blood count (CBC) with differential and platelets
- Serum chemistries: electrolytes, creatinine, urea, uric acid, bilirubin, AP, AST and / or ALT, LDH.
- Coagulation: PTT, quick, fibrinogen, ATIII
- Urine analysis
- Blood type
- Full history and clinical examination
- Vital signs
- Body height and body weight
- Performance status (ECOG)
- ECG
- Bone marrow aspirate and biopsy:
  - Cytogenetics
  - Cytomorphological examination including cytochemistry
  - Immunophenotyping
  - Molecular genetic analyses for the presence of Flt3-ITD, genomic ratio of the presence of Flt3-ITD vs. Flt3-WT, for nucleophosmin mutations, for the presence of Bcr-Abl, PML-RARα, AML1-ETO and CBFß-MYH11
  - Asservation of vital bone marrow cells (Ficoll-treated bone marrow), RNA, DNA and protein lysates, serum and plasma in a central tissue repository

6.1.2 Evaluations during treatment

(for full details about timepoints of evaluations please refer to 6.2)

- Control of CBC (with differential and platelets) weekly
- Bone marrow aspirate between day 24 and day 28 of each cycle
- Asservation of vital bone marrow cells (Ficoll-treated bone marrow), bone marrow RNA, DNA and protein lysates, serum and plasma in a central tissue repository.
- Control of coagulation and serum chemistries
- Performance status

- Physical examination
- Body weight
- Diagnostic procedures i.e. in case of severe infections will follow standard procedures
- Baseline evaluations will be repeated prior to each following course if necessary in the investigator's opinion
- ECG as often as necessary in the investigator's opinion

6.1.3 Evaluations in case of (suspected) relapse

- CBC (with differential and platelets)
- Bone marrow aspirate
- Asservation of vital bone marrow cells (Ficoll-treated bone marrow), bone marrow RNA, DNA and protein lysates, serum and plasma in a central tissue repository

6.1.4 Evaluations during observation of responding patients (CR, CRp, CRi)

Responding patients will be observed until death, or until one year after the start of therapy. The evaluations during observation include:

- In patients with relapse or patients refractory to treatment, patients will be monitored for new AEs / SAEs until 28 days after the last protocol treatment and for outcome of any AEs / SAEs (see chapter 7).
- CBC with differential monthly
- Bone marrow aspirate every three months and in case of suspected relapse
- one confirmatory bone marrow assessment including bone marrow trephine 4 weeks after the initial diagnosis of CR / CRp

6.1.5 Evaluations during follow-up in relapsed / refractory patients

- In patients with relapse or patients refractory to treatment, patients will be monitored for new AEs / SAEs until 28 days after the last protocol treatment and for outcome of any AEs / SAEs (see chapter 7).
- Patient's survival status will be monitored until one year after start of treatment.
- Additional follow-up visits will be performed at the investigator's discretion

6.2 Schedule of evaluations at baseline and during therapy

The schedule of evaluations before, during and after treatment are listed in table 2

Table 2. Listing of study evaluations

	Within 14 days prior to start of treatment	Days 5, 12, 19 ± 2 during therapy	Day 26 ± 2 during therapy	During observation in responding patients	End of study, suspected relapse
Informed consent	X				
Exclusion of intensive therapy	X				
Medical history	X				
Performance status and physical examination[a]	X		X	X[b] every 3 months	X
CBC (with differential and platelets)[a]	X	X	X	X monthly	X
Chemistry[a]	X	X	X		
Coagulation[a]	X		X		
Urinalysis[a]	X				
Bone marrow aspirate	X		X	X[b] every 3 months	X
Bone marrow biopsy[c]	X			X[b]	
Bone marrow and blood samples for exploratory research	X		X	X[b] every 3 months	X

[a] In addition, these evaluations should be repeated as often as necessary in the opinion of the treating physician

[b] Response confirmation including bone marrow biopsy one month after begin of observation period in responding patients, then bone marrow aspirate every 3 months or at suspected relapse

[c] Bone marrow biopsy only at initial diagnosis and at response confirmation 4 weeks after initial response; on other time points, only in case of dry tab

6.3 Central Bone marrow diagnostics and shipment modalities

The diagnosis of AML will be performed at the participating centers. For the central tissue and serum repository, bone marrow aspirate, bone marrow biopsy, peripheral blood and serum of each patient has to be sent to Dresden, Frankfurt or Münster at diagnosis and during follow-up.

6.3.1 Molecular genetic analyses

All molecular genetic analyses may be performed at the participating centers or at a laboratory of the investigators' choice.

Molecular genetic analysis has to include:

- determination of the presence or absence of Bcr-Abl, PML-RARα, AML-ETO, and CBFβ-MYH11 fusion transcripts as determined by qualitative or quantitative RT-PCR
- Nucleophosmin mutational status
- Determination of the presence or absence of Flt3-ITD and a quantification of the Flt3-ITD to Flt3-WT genomic ratio in case of the detection of Flt3-ITD

For a central molecular analysis, 5 ml heparinized bone marrow can be sent to the central laboratories in Frankfurt, Münster or Dresden listed below (6.3.2). Other laboratories may also be chosen.

6.3.2 Requested material and shipment modalities

At diagnosis and during follow-up bone marrow evaluations, please collect for central diagnostics and central tissue and serum repository:

**Central cytomorphological review (on request)**	5 ml bone marrow aspirate in EDTA<br>at least 4 unstained bone marrow smears<br>2 unstained peripheral blood smears
**Central immunophenotyping (on request)**	6-8 ml heparinized bone marrow
**Central molecular diagnosis[a] (on request)**	6-8 ml heparinized bone marrow
**Central histology[b] (mandatory)**	1 bone marrow biopsy, fixed in 'Kölner Lösung'
**Central cell repository (mandatory)**	2 x 6-8 ml heparinized bone marrow<br>20 ml heparinized peripheral blood<br>9 ml serum

[a] At diagnosis. During follow-up only in case of detection of a molecular marker at diagnosis

[b] At diagnosis and at the time of response confirmation one month after begin of observarion period in responding patients

The fixation solution ‘Kölner Lösung’ will be sent to the participating centers at the time of study initiation, every 4 weeks, and additionally upon request (phone: 0251/83-49963, or fax: 0251/83-47599).

Shipment is recommended from Monday through Thursday, if possible in the morning by express delivery or courier to the following addresses:

Universitätsklinikum<br>Carl Gustav Carus<br>Med. Klinik und Poliklinik I<br>Hämatol. Labor Haus 65 a<br>Fetscherstr. 74<br>01307 Dresden<br><br>Phone: 0351/458-4251<br>Fax: 0351/458-4367<br><br>For information on results, contact:<br><br>Prof. Dr. med. C. Thiede<br><br>*Phone: 0351/458-5628*	Universitätsklinikum Münster<br>Medizinische Klinik A<br>Labor für Spezielle Hämatologie<br>(Ebene 05)<br>Albert-Schweitzer-Straße 33<br>48149 Münster<br><br>Phone: 0251/83-47608<br>Fax: 0251/83-47633<br><br>For information on results, contact:<br><br>PD Dr. med. U. Krug<br>email: Utz.Krug@ukmuenster.de<br>*Phone: 0251/835-2995*	Universitätsklinikum Frankfurt<br>Medizinische Klinik II<br>Labor für Molekulare Diagnostik<br>Haus 33, UG, Raum 6<br>Theodor-Stern-Kai 7<br>60590 Frankfurt<br><br>Phone: 069/6301-83044<br>Fax: 069/6301-83046<br><br>For information on results, contact:<br><br>Dr. med. H. Pfeiffer<br>email: H.Pfeifer@em.uni-frankfurt.de<br>*Phone: 069/6301-83044*

6.3.3 Cytomorphologic examination, Cytochemistry, Histology and Immunohistochemistry

Initial leukemia diagnostics and differentiation are based on the morphology of peripheral blood and bone marrow smears as well as on cytochemical examinations. A Pappenheim staining, a peroxidase reaction and an esterase reaction are performed. Diagnosis and classification of the AML will be performed according to FAB criteria. These analyses will be performed in the local participating centers. If the bone marrow aspirate is not available, diagnosis will be based on the bone marrow biopsy. A central bone marrow biopsy diagnosis will be performed by the department of Pathology of the University Hospital of Muenster free of charge.

6.3.4 Immunphenotyping

An immunologic typing of the leukemia cells should be performed for all patients. These typings are performed at the local study centers. The immunophenotyping should contain the antigen CD56 and should be performed according to the proposals by the network of excellence “Acute and chronic leukemias”.

6.3.5 Central cytomorphology and immunophenotyping

The central study offices participate in round robin tests within the scope of the network of excellence for acute and chronic leukemias and offer reference laboratory diagnostics on request. In these cases, shipment of sufficient diagnostic material is required (for shipping modalities, see 6.3.2). For discussion of cytomorphology or immunophenotypic results, one of the persons below can be contacted:

Dr. S. Parmentier<br>PD Dr. F. Kroschinsky<br><br>Universitätsklinikum<br>Carl Gustav Carus<br>Med. Klinik und Poliklinik I<br>Hämatol. Labor Haus 65 a<br>Fetscherstr. 74<br>01307 Dresden<br><br>Phone: 0351/458-5627<br>resp. -4251<br>Fax: 0351/458-4367	PD Dr. Utz Krug<br>Prof. Dr. C. Müller-Tidow<br>Prof. Dr. T. Büchner<br>Universitätsklinikum Münster<br>Medizinische Klinik A<br>Labor für spezielle<br>Hämatologie (Ebene 05)<br>Albert-Schweitzer-Straße 33<br>48129 Münster<br><br>Phone: 0251/835-2995<br>Fax: 0251/835-2673<br>email:<br>Utz.Krug@ukmuenster.de	Dr. med. B. Steffen<br>Dr. med. U. Brunnberg<br>Dr. med. C. Brandts<br>Universitätsklinikum Frankfurt<br>Medizinische Klinik II<br>Labor für Molekulare Diagnostik<br>Haus 33, UG, Raum 6<br>Theodor-Stern-Kai 7<br>60590 Frankfurt<br><br>Phone: 069/6301-83044<br>Fax: 069/6301-83046

6.3.6 Cytogenetics

For each patient a cytogenetic examination has to be immediately performed at diagnosis. Chromosomal G-Banding will be performed. Fluorescent-in-situ-hybridizations will be performed if necessary. The cytogenetic examination will be organized by the local study centers.

Cytogenetic laboratories:

(Other laboratories may also be chosen .)

Dr. U. Pascheberg / W. Peter<br>Gemeinschaftspraxis für Laboratoriumsmed.Dr.<br>Eberhard und Partner<br>Brauhausstr. 4<br>44137 Dortmund<br><br>*Phone: 0231/9572-606*<br>*Fax: 0231/9572-636*	Dr. E. Krasemann<br>Labor Drs. med. Fenner<br>Abt. Humangenetik<br>Bergstr. 14<br>20095 Hamburg<br><br>*Phone: 040/30955-0*<br>*Fax: 040/30955-13*
Prof. Dr. Dr. H. Zankl / Dr. B. Thiele<br>Institut für Immunologie und Genetik<br>am Klinikum Kaiserslautern<br>Laborarztpraxis<br>Hellmut-Hartert-Str. 1<br>67613 Kaiserslautern<br><br>*Phone: 0631/316-700*<br>*Fax: 0631/316-7020 / 21*	Dr. rer. medic. B. Mohr<br>Universitätsklinikum Dresden<br>Med. Klinik und Poliklinik I<br>Haus 65 a<br>Fetscherstr. 74<br>01307 Dresden<br><br>*Phone: 0351/458-3377*<br>*Fax: 0351/458-4394*
Prof. Dr. Wieacker / Dr. S. Volpert<br>Institut für Humangenetik<br>Universitätsklinikum Münster<br>Vesaliusweg 12-14<br>48149 Münster<br><br>*Phone: 0251/83-55401*<br>*Fax: 0251/83-55431*	Prof. Dr. med. T. Haferlach /<br>PD Dr. med. C. Haferlach<br>MLL Münchner Leukämielabor GmbH<br>Max-Lebsche-Platz 31<br>81377 München<br><br>*Phone: 089/990-17-0*<br>*Fax: 089/990-17-111*

6.3.7 Exploratory research

For exploratory research purposes, an analysis of the bone marrow microvessel density in bone marrow biopsies at diagnosis and in remission, the expression levels and serum levels of VEGF,

VEGFR2, bFGF, FGFR, PDGFR, Src, and the the phosphorylation status of target genes (i.e. STAT5) will be determined at diagnosis and in remission. For this analysis the material listed above has to be sent to one of the three central laboratories Dresden, Münster or Frankfurt (see 6.3.2):

6.4 Duration of the study

Accrual time: 24 months	Treatment / observation[a]: 12 months

[a] In case of a relapse / treatment failure, a study exclusion or a refusal of informed consent, patients will be followed up for survival until one year after the start of the first induction cycle.

Assuming an accrual time of 24 months and a duration of treatment, observation or follow-up (of the last included patient) of approximately 12 months the duration of the study will be approximately 36 months.

6.5 End of study

The study will end at the time of data bank lock.

6.6 Criteria for removal from study / premature end of study

6.6.1 Individual reasons (Criteria for removal of patients)

Patients will be removed from the trial for the following reasons:

- drug-related toxicity
- Patient decision
- Incompliance

6.6.2 General reasons

The trial will be prematurely ended, if the safety committee recommends it for extensive toxicity.

7 ADVERSE EXPERIENCES

7.1 Definitions

7.1.1 Adverse Event (AE)

An adverse event is any untoward medical occurrence, including an exacerbation of pre-existing condition, in a patient or clinical trial subject administered an investigational medicinal product and which does not necessarily have a causal relationship with this treatment.

An adverse event (AE) can therefore be any unfavourable and unintended sign (including an abnormal laboratory finding), symptom, or disease temporally associated with the use of an investigational medicinal product, whether or not considered related to the investigational medicinal product.

Protocol-specific clarifications to this definition:

In order to monitor the safety of the trial participants throughout the trial, untoward medical occurrences between signature of the informed consent form and first administration of the investigational medicinal product also have to be documented as adverse events.

A pathological finding, improved or unchanged in comparison to baseline, does not constitute an adverse event.

Symptoms of the disease under study should not be classified as AEs as long as they are within the normal day-to-day fluctuation or expected progression of the disease unless having a fatal outcome.

Worsening of pre-existing conditions

A pre-existing condition present at baseline, which remains unchanged during the trial, does not need to be recorded as adverse event. Any worsening of any pre-existing baseline condition should be reported as an adverse event. Examples of worsening of a preexisting condition that should be recorded as an AE are given below;

- Worsening of condition meets the criteria for an SAE
- Action is taken with the investigational drug (i.e. dose is reduced or treatment is discontinued)
- Treatment is required (concomitant medication is added or changed)
- The investigator believes a patient has shown a clear deterioration from baseline symptoms

The reporting of abnormal laboratory values as adverse events should be avoided unless they lead to specific therapeutic consequences.

7.1.2 Adverse Reaction (AR)

An adverse reaction is any untoward and unintended response to an investigational medicinal product related to any dose administered.

All adverse events judged by either the reporting investigator or the sponsor as having a reasonable causal relationship to a medicinal product qualify as adverse reactions. The expression reasonable causal relationship means to convey in general that there is evidence or argument to suggest a causal relationship.

7.1.3 Serious Adverse Event (SAE) or Serious Adverse Reaction (SAR)

A serious adverse event or serious adverse reaction is any untoward medical occurrence or effect that at any dose

- results in death,
- is life-threatening,
- requires hospitalization or prolongation of existing inpatients' hospitalization,
- results in persistent or significant disability or incapacity,
- is a congenital abnormality or birth defect.

Comments:

- All deaths including death to disease progression up to and including 28 days after the last protocol treatment and death after the observational period (28 days after the last protocol treatment) which deemed to be related have to be reported immediately on an SAE form. The adverse event is the underlying event which caused the death.
- Life-threatening in the definition of a serious adverse event or serious adverse reaction refers to an event in which the subject was at risk of death at the time of event; it does not refer to an event which hypothetically might have caused death if it were more severe.
- Hospitalization means overnight admission.
- Hospitalization without underlying adverse event (AE) is not an SAE. Examples are:
    - Hospitalization for protocol procedures e.g. chemotherapy
    - Elective hospitalization for a pre-existing condition (i.e. a condition other than the indication for the chemotherapy) that has not worsened
    - Hospitalisation which was already planned at the beginning of the trial. Hospitalisation must have been reported at screening visit in the source data and have been performed as planned

    - Admission to a rehabilitation centre or hospice
    - Hospitalization for administrative or social reasons (e.g. due to anxiety but otherwise treatable on an outpatient basis).

- Congenital abnormality or birth defect: Fathering a child under BIBF 1120 is reportable as SAE in order to identify and follow-up on outcome of pregnancy and on any congenital abnormalities. The report should be made as soon as the investigator gets knowledge of the pregnancy. Follow-up of each pregnancy will be done using specific additional questionnaires supplied by the Safety Desk. Study participants will be informed about the necessity to follow up any pregnancy starting under study participation and are requested to consent into a follow-up of the course of any pregnancy in the patient information and informed consent.
- Medical judgment should be exercised in deciding whether an adverse event/reaction is serious in other situations. Important adverse events/ reactions that are not immediately life-threatening or do not result in death or hospitalization but may jeopardize the subject or may require intervention to prevent one of the other outcomes listed in the definition above, may also be considered serious.
- A new malignancy is a medically important condition and always considered as serious adverse event according to this protocol.

Protocol-specific events of special interest:

A hepatic injury defined by the following alterations of liver parameters is an event of special interest. For patients with normal AST / ALT and bilirubin at baseline, an elevation of AST and/or ALT above >3 fold ULN combined with an elevation of bilirubin above >2 fold ULN should be recorded as an SAE.

Protocol-specific exceptions for SAE reporting:

Leukemia-associated serious adverse events do not have to be reported as serious adverse events on this protocol as long as they are not fatal. These serious adverse events are restricted to the following events, only when considered leukemia-associated by the investigator: leukopenia, leukocytosis, neutropenia, anemia, thrombocytopenia, febrile neutropenia, neutropenic infections, thrombopenic bleedings.

Myelosuppression, thrombocytopenia, anemia and associated complications are expected events during leukemia therapy and are part of the treatment success (marrow emptying of leukemia cells). Therefore, myelosuppression-associated complications such as neutropenic fever, neutropenic

infections, thrombocytopenic bleeding, and related hospitalization will be recorded on the adverse event pages of the CRF as an adverse event and not reported on the SAE form.

7.2 Period of observation, documentation and reporting

All AEs, including SAEs, will be recorded from the time of signing the informed consent until 28 days after last protocol treatment including low-dose cytarabine. In case a patient leaves this protocol to receive another cytostatic or investigational therapy (that is for persistent or recurrent disease), the following modification applies: AEs and SAEs occurring within 28 days after last protocol treatment only have to be recorded on the present protocol when the investigator suspects the event to be related to the protocol treatment. Medical judgment should be used to determine the relationship, considering all relevant factors, including pattern of reaction, temporal relationship, de-challenge or re-challenge, confounding factors such as concomitant medication, concomitant diseases and relevant history. Assessment of causal relationship must be recorded for each adverse event

All AEs, including SAEs, have to be recorded on the appropriate adverse event pages in the CRF.

Documentation and assessment by the investigator includes:

- Diagnosis or Description of AE
- Date of onset and date of end of AE
- Seriousness (yes/no)
- Severity, graded according to the National Cancer Institute Common Terminology Criteria for Adverse Events (CTCAE) v 3.0 for Cancer Clinical Trials
- Causality (reasonable possibility/no reasonable possibility)
- Action taken with investigational medicinal drug (none/dose changed/drug temporarily withdrawn/drug permanently withdrawn)
- Outcome (recovered/resolved, recovering/resolving, not recovered/not resolved, recovered/resolved with sequelae, fatal, unknown)

If possible, a diagnosis rather than a list of signs, symptoms and laboratory abnormalities should be given.

All AEs, including SAE, must be followed up until the condition resolves or stabilizes. (For definition of resolution of an adverse event please see 5.3.4.1.)

All SAEs identified by the protocol as requiring immediate reporting must be reported on the SAE form and transmitted by fax to the Safety Desk within 1 business day of knowledge by the investigator. SAE’s will be reported to Boehringer Ingelheim by the sponsor within the format and the

timelines specified in the contract between sponsor and Boehringer Ingelheim. This applies regardless of severity (CTCAE v 3.0 grade) and whether or not the SAE is considered related to the use of study drug by the investigator. Personal data have to be replaced by the patient registration number before forwarding any information.

Where possible, a diagnosis rather than a list of symptoms should be given. The investigator is responsible for assessment of seriousness, severity (CTCAE v 3.0) and causality of the SAE. The SAE form should be completed with as much information as possible. The investigator should not wait for full details before making the initial report.

Safety-Desk-Contact
Zentrum für Klinische Studien (ZKS) Münster<br>Von-Esmarch-Str. 62<br>48129 Münster<br>Phone: 0251 83 57109<br>Fax: 0251 83 57112<br>E-Mail: mssd@ukmuenster.de

If the event is fatal or life threatening, the investigator must fax any relevant follow-up information of the reported SAE to the Safety Desk within 8 days from the initial report. In case of death, a pseudonymized copy of the autopsy protocol should be provided to the ZKS, if any. For SAEs, which are not fatal or life threatening, the investigator must fax follow-up information as soon as possible.

The investigator should answer any queries from the Safety Desk and Boehringer Ingelheim as soon as possible. In case of death, the investigator also has to supply the competent authority and the Ethics Committee with any details, if requested by them.

If the investigator suspects a reasonable causal relationship to the investigational medicinal product and/ or study design in an SAE occurring after the end of the reporting period, this event may also be reported to the Safety Desk. The investigator is explicitly encouraged to report late serious adverse reactions which are not yet listed in the Investigator's Brochure. Late SAEs do not have to be recorded on the adverse event page of the CRF. Reporting to the Safety Desk may be done on an SAE form or formless.

Assessment of serious adverse events by the sponsor

The Safety Desk will document each SAE, check it and query additionally required information. The Principal Investigator will review each SAE again for seriousness and relatedness.

Furthermore the Principal Investigator will assess whether an SAR is expected or unexpected according to the Investigator's Brochure and whether any SAE might influence the benefit-risk-ratio or require changes in the conduct of the trial.

“Unexpected” means that the nature, severity or outcome of the adverse reaction is not consistent with the applicable product information (Investigator’s Brochure).

- The term “severe” is often used to describe the intensity (severity) of a specific event. This is not the same as “serious,” which is based on patient/event outcome or action criteria.

Examples of unexpected events include:

- A more specific reaction than labelled (“acute renal failure” is a labelled AR, a new report of “interstitial nephritis” is more specific and therefore unexpected).
- An increase in the rate of occurrence of an expected AR, which is judged to be clinically important, is considered as unexpected.

A suspected unexpected serious adverse reaction (SUSAR) is a serious adverse reaction that has been judged to be unexpected.

Legal reporting requirements of the sponsor

It is the duty of the Safety Desk to ensure that Ethics Committee, competent authority and participating investigators are informed of all suspected unexpected serious adverse reactions (SUSARs) and all other relevant safety information in accordance with legal requirements.

It is the duty of the Safety Desk to inform the marketing authorization holder involved according to stipulation.

The Coordinating Investigator is responsible for the ongoing safety evaluation of the trial. The Safety Desk will inform the Principal Investigator immediately about any relevant safety information coming to its knowledge as will the Principal Investigator inform the Safety Desk. In case of other safety relevant issues (besides SUSARs) which require expedited reporting, the Safety Desk will support the Principal Investigator in submitting an appropriate report in due time.

The Principal Investigator is responsible for providing the updated benefit-risk assessment of the trial for the Annual Safety Report (Part 1 of the current report or corresponding parts of future reports according to legal requirements). The Safety Desk is responsible for preparing all other parts of the report, finalizing it and submitting it to the competent authority and ethics committee in due time.

The Safety Desk will provide information for the Safety Committee as requested (see also 9.1).

7.3 Warnings and Precautions

7.3.1 Investigational product (BIBF 1120)

7.3.1.1 Summary of known adverse drug reactions

Adverse reactions have been collected from phase I/II monotherapy studies with BIBF 1120 and combination studies of BIBF with cytotoxic agents. A summary of all adverse reactions is listed below [16].

Gastrointestinal adverse events

Frequent gastrointestinal adverse events included nausea (55%), diarrhea (43.9%), vomiting (39.8%), fatigue (24.8%), asthenia (15.0%) and abdominal pain (11.8%). Grade 3/4 adverse reactions of the above listed occurred in 0.7 to 3.3% in monotherapy studies and between 2.1 and 9.5% in the combination studies with Docetaxel / Prednisone, Carboplatin / Paclitaxel or Pemetrexed. All gastrointestinal adverse events were fully reversible.

Elevation of transaminases

While 10 – 15% of patients treated with doses 250mg of BIBF 1120 bidaily experienced a grade 3/4 transaminase elevation, no grade 3/4 transaminase elevation was observed at doses of 200 mg bidaily or lower. Transaminase elevations grade ≤ 2 were rarely seen at doses of 200 or 150 mg BIBF bidaily, either as monotherapy or in combination with chemotherapy. All transaminase elevations normalized after dose reduction or interruption of BIBF 1120.

Infections

Infectious complications were a frequent event both in the monotherapy and in the combination therapy. However, only few infections (app 4%) were considered drug related.

Thrombembolic and cardial events

The frequency of drug related thromboembolic events was low across all completed studies using BIBF 1120 monotherapy, and the frequency of thromboembolic adverse events regardless of relatedness did not seem to exceed the expected rate in the patients with advanced solid tumours.

Hypertension

Blood pressure elevation occurred in app. 10% of all patients, the vast majority with grade 1-2. Very rarely a grade 3/4 hypertension was seen, which suggests a relatively low hypertensive potential of BIBF 1120 as compared to other antiangiogenic compounds.

Dermatological events

Based on the current clinical data, the risk for light induced dermatological reactions is considered to be low.

Bleeding events

Events recorded bleeding events encoded the following: anemia, epistaxis, haemoptysis, hemorrhage, prolonged bleeding time and hematoma considered to be treatment-related occurred in 6% in the monotherapy and 13% in the combination therapy studies.

Further details of the side effect profile of BIBF 1120 can be found in the current version of the Investigator´s Brochure (IB). The IB is updated on a regular basis.

7.3.2 Cytarabine

For information of all known adverse drug reactions please refer to the 'Fachinformation Deutschland' in its latest version.

8 STUDY OUTCOME AND STATISTICAL ANALYSIS

8.1 Power and Sample Size Calculation

In phase II of the study the primary endpoint is the overall response rate (ORR) in the whole study population. In the primary analysis the following null hypothesis will be tested against a one-sided alternative on the significance level alpha=0.025.

H_0: ORR≤25% versus H_1: ORR>25%

The ORR under BIBF 1120 treatment is expected to depend on the absence or presence of a Flt3-mutation. In Flt3-mutated and Flt3-wildtype patients the expected ORR is ORR_{Flt3}=50% and ORR_{Flt3}=35%, respectively. In present data of a comparable population 13% of study patients showed a Flt3-mutation. Thus across all study patients the expected ORR amounts to ORR = 50%·0.13 + 35%·0.87 = 37%. A single stage design with fixed sample size was chosen. Given the above expected ORR=37%, in order to provide a 1-β=80% power of the primary hypothesis test, 111 evaluable patients are required to be included in the study, i.e. expectedly 14 Flt3-mutated and 97 Flt3-wildtype patients. 11 patients (10%) are anticipated not to be evaluable in the primary analysis due to missing data. Thus a total of 122 patients will be recruited into the phase II part of study.

Power calculations were performed using SAS version 9.2 (SAS Institute Inc., Cary, NC, USA).

8.2 Statistical Analysis Plan

The Statistical Analysis Plan (SAP) will be established without having insight into study data. In the SAP the statistical analyses are described in detail. The primary analysis will be performed using the one-sample (exact) Binomial test, applied according to the intention to treat principle (ITT). This means that every patient included into the study will be evaluated, even if he or she did not receive study medication, or any other violations of the study protocol occurred. Results of primary ITT analyses provide confirmatory statistical evidence. In addition to the ITT analysis a per protocol analysis (PP) will be performed. In the PP analysis only those patients are included who could be treated with full adherence to the protocol. Beyond the primary analysis, pre-specified analyses of secondary endpoints will be performed with appropriate α-adjustment due to multiplicity. Further exploratory analyses will be performed and interpreted according to the relatively low level of scientific evidence provided.

8.3 Number of patients

Under the above assumptions (see 8.1), total accrual to the controlled phase of this trial is planned for 122 patients.

8.4 Efficacy

Evaluation of bone marrow aspirate, peripheral blood counts and differentials are used to assess the efficacy of the study medication.

8.4.1 Response criteria

Response criteria are defined according to the Revised Recommendations of the International Working Group for Diagnosis, Standardization of Response Criteria, Treatment Outcomes, and Reporting Standards for Therapeutic Trials in Acute Myeloid Leukemia [18].

<u>Responding Patients:</u>

Morphologic complete remission (CR):

- Platelet count >100,000/µl
- Granulocyte count of >1,000/µl
- Bone marrow (aspirate with marrow spicules): < 5% blasts, Absence of Auer rods
- No evidence of persisting leukemia by flow cytometry (Sensitivity: 5%)
- Absence of extramedullary leukemia
- Transfusion independent stable hemoglobin value

Complete remission with incomplete platelet recovery (CRp):

- Granulocyte count of >1,000/µl
- Bone marrow (aspirate with marrow spicules): < 5% blasts, Absence of Auer rods
- No evidence of persisting leukemia by flow cytometry (Sensitivity: 5%)
- Absence of extramedullary leukemia
- Peripheral blood with no blast cells and less than 100,000/µl platelets

Complete remission with incomplete neutrophil recovery (CRi):

- Bone marrow (aspirate with marrow spicules): < 5% blasts, Absence of Auer rods
- No evidence of persisting leukemia by flow cytometry (Sensitivity: 5%)
- Absence of extramedullary leukemia
- Peripheral blood with no blast cells and less than 1,000/µl granulocytes irrespective of the platelet count

Cytogenetic Complete Response (CRc):

- Platelet count >100,000/µl
- Granulocyte count of >1,000/µl
- Bone marrow (aspirate with marrow spicules): < 5% blasts, Absence of Auer rods
- No evidence of persisting leukemia by flow cytometry (Sensitivity: 5%)
- Absence of extramedullary leukemia
- Transfusion independent stable hemoglobin value
- Normal cytogenetics (based on conventional banded studies and FISH)

Molecular Complete Response (CRm):

- Platelet count >100,000/µl
- Granulocyte count of >1,000/µl
- Bone marrow (aspirate with marrow spicules): < 5% blasts, Absence of Auer rods
- No evidence of persisting leukemia by flow cytometry (Sensitivity: 5%)
- Absence of extramedullary leukemia
- Transfusion independent hemoglobin value
- Normal cytogenetics (based on conventional banded studies and FISH)
- Molecularly negative (no detection of pre-treatment genetic markers with a methodology providing a sensitivity of at least $1{:}10^3$)

<u>Treatment failure:</u>

Partial Remission (PR):

- Platelet count >100,000/µl
- Granulocyte count of >1,000/µl
- Bone marrow (aspirate with marrow spicules): Decrease of at least 50% in the percentage of blasts to 5% - 25%

Resistant disease:

- Patient survives ≥ 7 days post Chemotherapy (CT); persistent AML in blood or bone marrow

Death in Aplasia:

- Patient survives ≥ 7 days post Chemotherapy (CT); death while cytopenic, with aplastic bone marrow

Indeterminate cause:

- Patients who die < 7 days post CT; Patients who die > 7 days post CT with no PB blasts, but bone marrow examination not performed or not evaluable; Patients who do not complete the first course of therapy

Morphologic relapse:

- Reappearance of blasts post CT in PB or bone marrow

8.5 Definition of Study Endpoints

Complete remission rate (CR rate):
Proportion of patients who achieve a complete remission (CR, as defined above) at any time point during study participation.

Overall response rate (ORR):
Proportion of patients who achieve either a complete remission (CR), a CRp or a CRi, as defined above, at any time point during study participation.

Overall survival (OS):

Time interval from day 1 of study treatment to the day of death. For a patient who is not known to have died by the end of follow-up, observation of OS will be censored on the date the patient was last known to be alive.

Relapse-free survival (RFS):

Time interval from the first day a leukemia-free state is achieved in patients who achieve a CR until relapse or death from any course, whatever occurs first. For a patient who is not known to have died or have relapsed by the end of follow-up, observation of RFS will be censored on the date the patient was last known to be alive and in CR.

9 QUALITY ASSURANCE

9.1 Data Monitoring Committee

The Data Monitoring Committee of this trial will consist of three members of the scientific community not involved in this trial. The committee will commence regularly (by phone conference) with the Principal Investigator of the trial and review all serious adverse events (SAE) and suspected unexpected severe adverse reactions (SUSARs) and has the right to request any information to be able to assess the safety of the trial participants. The Safety committee will give recommendations about the continuation of the trial and/or about necessary trial amendments.

9.2 Reference Laboratories

9.2.1 Reference diagnostics for cytomorphology

As stated above, a central review of selected cases will be performed in one of the three diagnostic reference centers.

Dr. S. Parmentier<br>Dr. PD Dr. F. Kroschinsky<br>Medizinische Klinik und Poliklinik I<br>Universitätsklinikum Carl Gustav Carus an der Technischen Universität Dresden<br>Hämatologisches Labor Haus 65a<br>Fetscherstr. 74<br>01307 Dresden<br><br>Phone: 0351/458-5627<br>resp. -4251<br>Fax: 0351/458-4367	PD Dr. med. Utz Krug<br>Prof. Dr. med. C. Müller-Tidow<br>Prof. Dr. med. T. Büchner<br>Universitätsklinikum Münster<br>Medizinische Klinik A<br>Labor für spezielle Hämatologie<br>Albert-Schweitzer-Straße 33<br>48129 Münster<br><br>Phone: 0251/835-2995<br>Fax: 0251/835-2673<br>email: Utz.Krug@ukmuenster.de	Dr. med. B. Steffen<br>Dr. med. U. Brunnberg<br>Dr. med. C. Brandts<br>Universitätsklinikum Frankfurt<br>Medizinische Klinik II<br>Labor für Molekulare Diagnostik<br>Haus 33, UG, Raum 6<br>Theodor-Stern-Kai 7<br>60590 Frankfurt<br><br>Phone: 069/6301-83044<br>Fax: 069/6301-83046

9.3 Monitoring

Before the beginning of the study there will be a central meeting for all investigators from all participating centers (initiation meeting). At this meeting there will be a central training concerning all the relevant details of the study.

During the phase I part of the study, 100% of the patients will be monitored.

During the course of the study each participating center will be visited for monitoring one to four times. During each of these visits, source data verification will be performed on the basis of a pre-

specified sampling plan. The ZKS Muenster will generate this plan. Furthermore, at these visits problematic cases as specified by the principal investigator will be discussed. Study centers can be selected for additional monitoring visits during the course of the study as judged necessary.

At the end of the study there may be a special close out visit for each center.

9.4 Direct access to source data - documents

The investigator / institution will permit trial-related monitoring, audits, IRB / IEC review and regulatory inspection, providing direct access to all related source data / documents. CRFs and all source documents, including progress notes and copies of laboratory and medical test results must be available at all times for review by the sponsor's clinical trial monitor, auditor and inspection by health authorities.

9.5 Data Management

For data management, an electronic database will be generated at the ZKS Muenster. The database will be equipped with an audit trail that allows for a full follow-up of the history of each item.

Data capture of the study is paper based using pre-specified case report forms (CRF). The CRF will be completed and sent to the ZKS within 6 weeks. At the ZKS Muenster, the incoming CRFs will be assessed for plausibility. The ZKS will try to resolve possible implausibility by queries to the responsible investigator at the involved center. The investigator has to respond by a signed written statement to be sent to the ZKS Muenster.

Further data processing including data entry and biometrical analysis will be done at the Institute of Biostatistics and Clinical Research of the university hospital of Münster (IBCR) and at the Zentrum für klinische Studien (ZKS). This will be done in close cooperation with the coordinating investigator.

10 INVESTIGATOR’S RESPONSIBILITIES, ETHICAL CONSIDERATIONS, CONFIDENTIALITY, PUBLICATION AND INSURANCE

10.1 Investigator’s responsibilities

The Principal Investigator has more than two years experience in the conduct of clinical trials.

10.1.1 Declaration of Helsinki and GCP compliance

The Investigator undertakes to perform the study in accordance with the Declaration of Helsinki (in its latest version Tokio, 2004) and the ICH Guidelines in Good Clinical Practice as well as with the applicable regulatory requirements.

10.1.2 Protocol adherence

The Investigator must adhere to the protocol as detailed in this document. The Investigator will be responsible for enrolling only those patients who have met protocol eligibility criteria.

10.1.3 Documentation and retention of records

10.1.3.1 Case Report Forms (CRFs)

The Investigator is responsible for maintaining adequate and accurate CRFs which have been designed to record all observations and other data pertinent to the clinical investigation. CRFs should be filled out completely by the Investigator or delegate as stated in the Site Delegation List. All CRFs should be completed in a neat, legible manner to ensure accurate interpretation of the data; a black ball-point pen should be used to ensure the clarity of reproduced copies of all CRFs. Corrections should be made by lining out the entry to be corrected by a single line and dating and signing the corrected entry. CRFs should be completed and sent to the ZKS within 6 weeks.

As described in the ICH GCP Guidelines (E6), ‘essential documents’, including CRFs, source documents, consent forms, laboratory test results, and medication inventory records, will be archived by the Investigator according to the current rules and regulations.

10.1.3.2 Recording, access, and retention of Source Data

Source documents are defined as any document where the information is collected during the study procedures for a specific subject. Source documents can be patient’s medical file, appointment books, original laboratory reports, X-rays, Investigator or nurses notes, etc. Source data to be collected during this study will include, but is not restricted to: patient’s medical file, original laboratory reports, histology, and pathology reports.

10.1.3.3 Storage of Data related to the clinical trial

All data relating to the clinical study, including the investigator file, CRFs and source data must be stored by the investigator for at least 15 years after the clinical study is finished. Clinical study records should not be destroyed without prior written agreement between the sponsor and the investigator.

10.1.4 Competent local authorities

It is the responsibility of the investigator to notify the competent local authority about the conduct of this trial before starting recruitment. The investigator shall inform the competent local authority within 90 days of termination of the clinical trial. Where the clinical trial has been suspended or interrupted by the sponsor, notification shall take place within 15 days, giving the reasons for suspension or interruption.

10.2 Ethical considerations

10.2.1 Institutional Review Board / Independent Ethics Committee approval

It is the responsibility of the investigator to provide all requested information about qualification of the respective trial site and trial staff to the sponsor. The sponsor will submit the application to the IRB/IEC.

The trial may only be conducted as approved by the Ethics committee and the competent authority. Amendments may only be implemented after approval. Additional trial sites may only recruit patients after the sponsor obtained approval for the site.

In compliance with European regulations/ICH-GCP Guidelines, it is required that the investigator and institution permit authorized representatives of the study central office and the regulatory agency(s) direct access to review the subject's original medical records for verification of study-related procedures and data. Direct access includes examining, analyzing, verifying, and reproducing any records and reports that are important to the evaluation of the study. The investigator is responsible for giving any requested support for any monitoring, inspection or audit visit. The investigator has to be available during these visits.

10.2.2 Informed Consent

It is the responsibility of the Investigator to obtain written Informed Consent from patients. Each patient or the patient's legal guardian is requested to sign the Patient Information and Consent Form after the patient has received written information and an explanation of what the study involves (i.e., the objectives, potential benefits and risk, inconveniences and the patient's rights and responsibilities). A copy of the patient Information and signed Consent Form must be given to the patient or the patient's legal guardian.

10.3 Confidentiality

The investigator must ensure that the patient's encryption is maintained. On the CRFs or other documents submitted to the study central office, subjects should be identified by a subject study number only. Documents that are not for submission to the study central office (e.g., signed informed consent forms) should be kept in strict confidence by the investigator. Investigator has to maintain a patient identification list ensuring patient identification by the registration number.

10.4 Publication

The findings from this clinical study should be published in a reputable peer-reviewed medical journal in accordance with basic ethical principles, including preservation of the accuracy of the results and making both positive and negative results publicly available. Authorship will be credited in accordance with ICMJE guidelines (www.icmje.org).

10.5 Insurance

For all patients in this trial, the sponsor has contracted an insurance covering possible damage to the patients at the Gerling-Konzern Allgemeine Versicherungs-AG. The insurance is part of the general insurance agreement between the Universitätsklinikum Münster AöR, Domagkstraße 5, 48149 Münster and the Gerling-Konzern, Police No.: ausstehend.

Adress of the Insurance Company:

Gerling Vertrieb Deutschland GmbH

Regionalzentrum West
Prinzenallee 21
40549 Düsseldorf
Telefon 0211 4956-0
Fax 0211 4956-487

11 References

1 Buchner T, Berdel WE, Schoch C, Haferlach T, Serve HL, et al. Double induction containing either two courses or one course of high-dose cytarabine plus mitoxantrone and postremission therapy by either autologous stem-cell transplantation or by prolonged maintenance for acute myeloid leukemia. J Clin Oncol 2006;24:2480-2489.

2 Burnett AK, Milligan D, Prentice AG, Goldstone AH, McMullin MF, et al. A comparison of low-dose cytarabine and hydroxyurea with or without all-trans retinoic acid for acute myeloid leukemia and high-risk myelodysplastic syndrome in patients not considered fit for intensive treatment. Cancer 2007;109:1114-1124.

3 Krug U, Serve H, Muller-Tidow C, Mesters RM, Steffen B, et al. New molecular therapy targets in acute myeloid leukemia. Recent Results Cancer Res 2007;176:243-262.

4 Deguchi K, Gilliland DG. Cooperativity between mutations in tyrosine kinases and in hematopoietic transcription factors in AML. Leukemia 2002;16:740-744.

5 Schnittger S, Schoch C, Dugas M, Kern W, Staib P, et al. Analysis of FLT3 length mutations in 1003 patients with acute myeloid leukemia: correlation to cytogenetics, FAB subtype, and prognosis in the AMLCG study and usefulness as a marker for the detection of minimal residual disease. Blood 2002;100:59-66.

6 Thiede C, Steudel C, Mohr B, Schaich M, Schakel U, et al. Analysis of FLT3-activating mutations in 979 patients with acute myelogenous leukemia: association with FAB subtypes and identification of subgroups with poor prognosis. Blood 2002;99:4326-4335.

7 Bos JL, Verlaan-de Vries M, van der Eb AJ, Janssen JW, Delwel R, et al. Mutations in N-ras predominate in acute myeloid leukemia. Blood 1987;69:1237-1241.

8 Hussong JW, Rodgers GM, Shami PJ. Evidence of increased angiogenesis in patients with acute myeloid leukemia. Blood 2000;95:309-313.

9 Padro T, Ruiz S, Bieker R, Burger H, Steins M, et al. Increased angiogenesis in the bone marrow of patients with acute myeloid leukemia. Blood 2000;95:2637-2644.

10 Fiedler W, Graeven U, Ergun S, Verago S, Kilic N, et al. Vascular endothelial growth factor, a possible paracrine growth factor in human acute myeloid leukemia. Blood 1997;89:1870-1875.

11 Padro T, Bieker R, Ruiz S, Steins M, Retzlaff S, et al. Overexpression of vascular endothelial growth factor (VEGF) and its cellular receptor KDR (VEGFR-2) in the bone marrow of patients with acute myeloid leukemia. Leukemia 2002;16:1302-1310.

12 Bieker R, Padro T, Kramer J, Steins M, Kessler T, et al. Overexpression of basic fibroblast growth factor and autocrine stimulation in acute myeloid leukemia. Cancer Res 2003;63:7241-7246.

13 Foss B, Ulvestad E, Bruserud O. Platelet-derived growth factor (PDGF) in human acute myelogenous leukemia: PDGF receptor expression, endogenous PDGF release and responsiveness to exogenous PDGF isoforms by in vitro cultured acute myelogenous leukemia blasts. Eur J Haematol 2001;67:267-278.

14 Kulimova E, Oelmann E, Bisping G, Kienast J, Mesters RM, et al. Growth inhibition and induction of apoptosis in acute myeloid leukemia cells by new indolinone derivatives targeting fibroblast growth factor, platelet-derived growth factor, and vascular endothelial growth factor receptors. Mol Cancer Ther 2006;5:3105-3112.

15 Ozawa Y, Williams AH, Estes ML, Matsushita N, Boschelli F, et al. Src family kinases promote AML cell survival through activation of signal transducers and activators of transcription (STAT). Leuk Res 2008;32:893-903.

16 BIBF 1120 Investigator's Brochure. current version. Boehringer Ingelheim.

17 Fenaux P, Mufti GJ, Hellstrom-Lindberg E, Santini V, Gattermann N, et al. Azacitidine prolongs overall survival compared with conventional care regimens in elderly patients with low bone marrow blast count acute myeloid leukemia. J Clin Oncol 28:562-569.

18 Cheson BD, Bennett JM, Kopecky KJ, Buchner T, Willman CL, et al. Revised recommendations of the International Working Group for Diagnosis, Standardization of Response Criteria, Treatment Outcomes, and Reporting Standards for Therapeutic Trials in Acute Myeloid Leukemia. J Clin Oncol 2003;21:4642-4649.

12 Appendices

12.1 Synopsis of the study (in German)

Studientitel	Eine einarmige, multizentrische Studie zur Erfassung der Effektivität der Gabe von BIBF 1120 zusätzlich zur niedrigdosiertem Cytarabin bei älteren Patienten mit nicht intensiv behandelbarer neudiagnostizierter AML
Klinische Phase	I/II
Primäres Studienziel	Phase I: Ermittlung der Sicherheit und Verträglichkeit der Kombination von BIBF 1120 mit niedrigdosiertem Cytarabin<br>Phase II: Ermittlung der Gesamtansprechrate (CR, CRp und CRi) der Kombinationstherapie
Sekundäre Studienziele (Phase II)	• Vergleich der Gesamtansprechraten von Patienten mit Flt3-mutierter AML und Patienten mit Flt3 Wildtyp AML sowie der Gesamtansprechraten innerhalb der verschiedenen zytogenetischen Risikogruppen<br>• CR-Rate in der gesamten Studiengruppe<br>• Vergleich der CR-Raten von Patienten mit Flt3-mutierter AML und Patienten mit Flt3 Wildtyp AML sowie innerhalb der verschiedenen zytogenetischen Risikogruppen<br>• Ermittlung des Ein-Jahres Gesamtüberlebens der gesamten Studienpopulation<br>• Vergleich der Ein-Jahres Gesamtüberlebenszeiten von Patienten mit Flt3-mutierter AML und Patienten mit Flt3 Wildtyp AML sowie innerhalb der verschiedenen zytogenetischen Risikogruppen<br>• Ermittlung des medianen leukämiefreien Überlebens der Patienten mit Ansprechen auf die Studientherapie<br>• Ermittlung der Zeit bis zum Erreichen eines Therapieansprechens (CR, CRp oder CRi) bei den Patienten mit Ansprechen auf die Studientherapie<br>• Ermittlung der Toxizität in der gesamten Studienpopulation<br>• Evaluation von Biomarkern bzgl. der Voraussage des Krankheitsverlaufs und des Therapieansprechens

Studiendesign	Prospektive, einarmige, multizentrische Phase I/II Studie<br><br>Alle Patienten erhalten eine Induktionstherapie mit einer Kombination aus subkutenem Cytarabin und oralem BIBF.<br><br>Phase I:<br>In der Phase I findet eine Dosisfindung nach einem klassischen 3+3-Design statt. Die verwendeten Dosisstufen von BIBF 1120 sind wie folgt vordefiniert:<br>• Dosisstufe 1: 100 mg zweimal tgl.<br>• Dosisstufe 2: 150 mg zweimal tgl.<br>• Dosisstufe 3: 200 mg zweimal tgl.<br><br>Phase II:<br>In der Phase II kommt die in der Phase I festgelegte Dosisstufe von BIBF 1120 zur Verwendung.<br><br>Therapiedurchführung:<br>AraC 2 x 20 mg s.c. Tag 1-10<br>BIBF 1120 2 x Dosisstufe p.o. Tag 1-28<br>*Wdh. für bis zu sechs Zyklen jeweils an Tag 29 bis zum Erreichen einer CR, CRp oder CRi, oder bis zum Auftreten intolerabler Toxizität oder einer signifikanten Erkankungsprogression,.*
Patientenzahl	9-12 (Phase I) bzw. 122 (Phase II)
Randomisierung	Die Randomisierung erfolgt über das Institut für Biostatistik und klinische Forschung (IBKF) der WWU Münster. (Tel.-Nr.: 0251/8355272, Fax: 0251/83555277)
Einschlusskriterien	• Pat. mit neu diagnostizierter AML (ausser APL) nach WHO und/oder FAB-Klassifikation, auch Pat. mit AML aus MDS oder anderer sekundärer AML, welche sich nicht für eine intensive Induktionstherapie qualifizieren oder diese verweigern<br>• Für Phase I zusätzlich auch Patienten mit refraktärer oder rezidivierter AML (ausser APL), welche sich nicht für eine intensive Salvage-Therapie qualifizieren oder diese verweigern<br>• Blastenanteil im Knochenmark > 20% bzw. >30% der nichterythropoetischen Zellen bei AML FAB M6. Bei Patienten mit bis zu 30% KM-Blasten sollte eine hypomethylierende Therapie erwogen werden.<br>• Alter ≥ 60 Jahre<br>• Unterschriebene Einverständniserklärung<br>• Adäquate Empfängnisverhütung für männliche Patienten

Ausschlusskriterien	• Patienten mit bis zu 30% Knochenmarkblasten, welche sich für eine hypomethylierende Therapie qualifizieren und in diese einwilligen<br>• Patienten, die für eine Standardchemotherapie qualifizieren und in diese einwilligen<br>• Bekannte ZNS-Beteiligung der AML<br>• Transaminasenerhöhung auf ≥ 2.5 x oberer Normwert, sofern nicht durch leukämische Infiltration erklärt<br>• Bekannte chronisch-aktive Hepatitis C oder akute Hepatitis<br>• Chronisch eingeschränkte Nierenfunktion mit einer Kreatinin-Clearance von < 30 ml/min<br>• Unter adäquater medikamentöser Therapie unkontrollierter arterieller Hypertonus mit Ruhe-RR-Werten > 160 mmHg systolisch oder > 95 mmHg diastolisch<br>• Schweres Trauma oder grosser chirurgischer Eingriff innerhalb 4 Wochen vor Aufnahme in die Studie<br>• Schwere chronische nichtheilende Wunden, Ulzera oder Frakturen<br>• Unkontrollierte aktive Infektion<br>• Schwere Begleiterkrankungen (inkl. Zweitmalignome), welche aufgrund der bestehenden Lebenserwartung nach Einschätzung des Prüfarztes wahrscheinlich die Studienendpunkte beeinflussen<br>• Unverträglichkeit gegen Cytarabin, mit Ausnahme von Medikamentenfieber und Exanthem<br>• Nur Phase II: Vorbehandlung der AML mit Ausnahme von Hydoxyurea bis 24h vor der ersten Studienmedikation<br>• Nur Phase II: vorangegangene Therapie mit Tyrosinkinaseinhibitoren oder Angiogenese-Inhibitoren<br>• Parallele Teilnahme an einer klinischen Studie für die gleiche Indikation. Bei Patienten mit einer experimentellen Therapie oder Studienteilnahme innerhalb 4 Wochen vor Einschluß in die Studie muß die Eligibilität vor Studieneinschluß mit der Studienzentrale geklärt werden<br>• Jeder Begleitzustand, welcher nach Einschätzung des Prüfarztes eine Studienteilnahme nicht ratsam erscheinen lässt oder die protokollgemässe Studiendurchführung behindert

12.2 Performance Status

ECOG		Karnofsky	
Score	Description	Score	Description
0	Fully active, able to carry on all pre-disease performance without restriction.	100	Normal, no complaints, no evidence of disease.
		90	Able to carry on normal activity; minor signs or symptoms of disease.
1	Restricted in physically strenuous activity but ambulatory and able to carry out work of a light or sedentary nature, e.g., light housework, office work.	80	Normal activity with effort; some signs or symptoms of disease.
		70	Cares for self, unable to carry on normal activity or do active work.
2	Ambulatory and capable of all self care but unable to carry out any work activities. Up and about more than 50% of waking hours.	60	Requires occasional assistance, but is able to care for most of his/her needs.
		50	Requires considerable assistance and frequent medical care.
3	Capable of only limited self care, confined to bed or chair more than 50% of waking hours.	40	Disabled, requires special care and assistance.
		30	Severely disabled, hospitalization indicated. Death not imminent.
4	Completely disabled. Cannot carry on any self care. Totally confined to bed or chair.	20	Very sick, hospitalization indicated. Death not imminent.
		10	Moribund, fatal processes progressing rapidly.
