# Supplementary material for: A Phase I Dose Escalation Study of the Triple Angiokinase Inhibitor Nintedanib Combined with Low-Dose Cytarabine in Elderly Patients with Acute Myeloid Leukemia
Source: PLoS One. 2016 Oct 7;11(10):e0164499. doi: 10.1371/journal.pone.0164499 (PMC5055288; doi:10.1371/journal.pone.0164499)
Supplement: S1 Table — (PDF) [file pone.0164499.s005.pdf]

MedDRA version 15.1		CTCAE grade					
System Organ Class	Preferred Term	All Grades	Grade 1	Grade 2	Grade 3	Grade 4	Grade 5
Blood and lymphatic system disorders	Febrile neutropenia	4	0	1	3	0	0
	Lymph node pain	1	1	0	0	0	0
	Thrombocytopenia	1	0	0	0	1	0
**Sub-total number of AEs**		**6**	**1**	**1**	**3**	**1**	**0**
Cardiac disorders	Atrial flutter	2	0	0	2	0	0
	Tachycardia	1	0	1	0	0	0
	Tachycardia paroxysmal	1	0	1	0	0	0
**Sub-total number of AEs**		**4**	**0**	**2**	**2**	**0**	**0**
Gastrointestinal disorders	Abdominal distension	2	2	0	0	0	0
	Abdominal pain	2	2	0	0	0	0
	Abdominal pain upper	3	1	2	0	0	0
	Anal fissure	2	1	1	0	0	0
	Constipation	4	3	1	0	0	0
	Diarrhoea	14	11	1	2	0	0
	Dry mouth	3	3	0	0	0	0
	Eructation	1	1	0	0	0	0
	Gingival bleeding	1	1	0	0	0	0
	Mouth haemorrhage	3	3	0	0	0	0
	Nausea	10	8	2	0	0	0
	Oral mucosal erythema	1	1	0	0	0	0
	Oral pain	1	1	0	0	0	0
	Proctalgia	1	0	0	1	0	0
	Rectal haemorrhage	1	1	0	0	0	0
	Vomiting	11	9	2	0	0	0
**Sub-total number of AEs**		**60**	**48**	**9**	**3**	**0**	**0**
General disorders and administration site conditions	Asthenia	4	4	0	0	0	0
	Chest pain	1	1	0	0	0	0

MedDRA version 15.1		CTCAE grade					
**System Organ Class**	**Preferred Term**	**All Grades**	**Grade 1**	**Grade 2**	**Grade 3**	**Grade 4**	**Grade 5**
	Chills	2	2	0	0	0	0
	Fatigue	1	1	0	0	0	0
	Oedema peripheral	5	4	1	0	0	0
	Pyrexia	6	4	2	0	0	0
**Sub-total number of AEs**		**19**	**16**	**3**	**0**	**0**	**0**
Infections and infestations	Gastrointestinal infection	1	0	0	0	0	1
	Nasopharyngitis	1	1	0	0	0	0
	Oral herpes	1	1	0	0	0	0
	Paronychia	1	1	0	0	0	0
	Pneumonia	1	0	1	0	0	0
	Soft tissue infection	1	1	0	0	0	0
**Sub-total number of AEs**		**6**	**4**	**1**	**0**	**0**	**1**
Injury, poisoning and procedural complications	Periorbital haematoma	1	1	0	0	0	0
**Sub-total number of AEs**		**1**	**1**	**0**	**0**	**0**	**0**
Investigations	Blood alkaline phosphatase increased	3	3	0	0	0	0
	Blood bilirubin increased	2	1	1	0	0	0
	Blood creatinine increased	1	0	0	1	0	0
	Blood urea increased	1	0	0	1	0	0
	Blood uric acid increased	1	1	0	0	0	0
	Heart rate irregular	1	1	0	0	0	0
	Oxygen saturation decreased	1	1	0	0	0	0
**Sub-total number of AEs**		**10**	**7**	**1**	**2**	**0**	**0**
Metabolism and nutrition disorders	Decreased appetite	4	4	0	0	0	0
	Dehydration	1	0	0	1	0	0
	Hypercalcaemia	1	0	0	0	0	1
	Hyperuricaemia	1	0	0	1	0	0

MedDRA version 15.1		CTCAE grade					
**System Organ Class**	**Preferred Term**	**All Grades**	**Grade 1**	**Grade 2**	**Grade 3**	**Grade 4**	**Grade 5**
**Sub-total number of AEs**		**7**	**4**	**0**	**2**	**0**	**1**
Musculoskeletal and connective tissue disorders	Arthralgia	3	2	1	0	0	0
	Back pain	3	2	1	0	0	0
	Joint swelling	1	1	0	0	0	0
	Muscular weakness	1	1	0	0	0	0
	Musculoskeletal chest pain	1	1	0	0	0	0
	Musculoskeletal pain	1	1	0	0	0	0
	Pain in extremity	2	2	0	0	0	0
**Sub-total number of AEs**		**12**	**10**	**2**	**0**	**0**	**0**
Neoplasms benign, malignant and unspecified (incl cysts and polyps)	Acute myeloid leukaemia	1	0	0	0	0	1
**Sub-total number of AEs**		**1**	**0**	**0**	**0**	**0**	**1**
Nervous system disorders	Dizziness	1	1	0	0	0	0
	Dysgeusia	1	1	0	0	0	0
	Headache	2	2	0	0	0	0
	Hypotonia	2	2	0	0	0	0
	Paraesthesia	2	2	0	0	0	0
	Tremor	1	1	0	0	0	0
**Sub-total number of AEs**		**9**	**9**	**0**	**0**	**0**	**0**
Psychiatric disorders	Disorientation	1	0	1	0	0	0
	Restlessness	4	3	1	0	0	0
**Sub-total number of AEs**		**5**	**3**	**2**	**0**	**0**	**0**
Renal and urinary disorders	Pollakiuria	1	1	0	0	0	0
	Renal failure acute	1	0	0	1	0	0
**Sub-total number of AEs**		**2**	**1**	**0**	**1**	**0**	**0**
Respiratory, thoracic and mediastinal disorders	Cough	2	1	1	0	0	0
	Dyspnoea	3	2	1	0	0	0

MedDRA version 15.1		CTCAE grade					
**System Organ Class**	**Preferred Term**	**All Grades**	**Grade 1**	**Grade 2**	**Grade 3**	**Grade 4**	**Grade 5**
	Dyspnoea exertional	1	0	1	0	0	0
	Epistaxis	3	1	2	0	0	0
	Oropharyngeal pain	1	1	0	0	0	0
	Pharyngeal erythema	1	1	0	0	0	0
**Sub-total number of AEs**		**11**	**6**	**5**	**0**	**0**	**0**
Skin and subcutaneous tissue disorders	Dry skin	2	2	0	0	0	0
	Night sweats	1	1	0	0	0	0
	Petechiae	1	1	0	0	0	0
	Rash	1	1	0	0	0	0
	Skin ulcer	1	1	0	0	0	0
**Sub-total number of AEs**		**6**	**6**	**0**	**0**	**0**	**0**
Vascular disorders	Haematoma	1	1	0	0	0	0
	Hypertension	1	1	0	0	0	0
	Intra-abdominal haematoma	1	1	0	0	0	0
	Peripheral coldness	1	1	0	0	0	0
**Sub-total number of AEs**		**4**	**4**	**0**	**0**	**0**	**0**
**Total number of AEs**		**163**	**120**	**26**	**13**	**1**	**3**
